# Supplementary material for: Computationally efficient mechanism discovery for cell invasion with uncertainty quantification
Source: PLoS Comput Biol. 2022 Nov 16;18(11):e1010599. doi: 10.1371/journal.pcbi.1010599 (PMC9710850; doi:10.1371/journal.pcbi.1010599)
Supplement: S1 Document — S1 Text. Simulation studies. Synthetic data explorations to demonstrate the veracity of our methods. S1 Fig A. Simulated data and fitted Gaussian process. Simulated data according to the model in S1 Eq B with D(u; β) = β1 and R(u; γ) = γ1u(1−u/K), with β1 = 301 μm2 and γ1 = 0.044 h−1. The lines and shaded regions represent a Gaussian process fit to the spatiotemporal data. S1 Fig B. Initial simulation results. Initial simulation results for the data from model in S1 Eq B where R(u; γ) = γ1u(1−u/K) and we use the misspecified nonlinear diffusivity model D(u; γ) = β1 + β2(u/K). In the density plots, the dashed red vertical line is at the position of the true parameter value, and in the curve plots the dashed red curve shows the true mechanisms. S1 Fig C. Final simulation results. Final simulation results after re-scaling for the data from the model in S1 Eq 5 where R(u; γ) = γ1u(1−u/K) and we use the misspecified nonlinear diffusivity model D(u; γ) = β1 + β2(u/K). The re-scaling used is given in S1 Eq C. In the density plots, the dashed red vertical line is at the position of the true parameter value, and in the curve plots the dashed red curve shows the true mechanisms. S1 Fig D. Initial simulation results. Initial simulation results for the data from model in S1 Eq B where R(u; γ) = γ1u(1−u/K) and we use the correctly specified model D(u; γ) = β1. In the density plots, the dashed red vertical line is at the position of the true parameter value, and in the curve plots the dashed red curve shows the true mechanisms. S1 Fig E. Final simulation results. Final simulation results after re-scaling for the data from the model in S1 Eq B where R(u; γ) = γ1u(1−u/K) and we use the correctly specified nonlinear diffusivity model D(u; γ) = β1. The rescaling used is β1=300β^1 and γ1=0.044γ^1. In the density plots, the dashed red vertical line is at the position of the true parameter value, and in the curve plots the dashed red curve shows the true mechanism. S1 Table A. Results f [file pcbi.1010599.s001.pdf]

Computationally efficient mechanism discovery for cell invasion with uncertainty quantification

Daniel J. VandenHeuvel[1], Christopher Drovandi[1], and Matthew J. Simpson[1]

[1]School of Mathematical Sciences, Queensland University of Technology, Brisbane, Queensland, Australia

October 2, 2022

In this document, all section, table, and equation numbers without the S-prefix refer to the main manuscript.

S1 Simulation studies

In this section we describe two simulation studies used for testing our results. The models will all take the form

$$\frac{\partial u}{\partial t} = T(t;\boldsymbol{\alpha})\left[\frac{\partial}{\partial x}\left(D(u;\boldsymbol{\beta})\frac{\partial u}{\partial x}\right) + R(u;\boldsymbol{\gamma})\right]. \tag{A}$$

To simulate data, we start with the data from Jin et al. [1] with an initial density of 10,000 cells per well, taking only the data at $t = 0$. We then define the grid $x_j = x_1 + (j-1)\Delta x$, where $\Delta x = (x_{N_p} - x_1)/(N_p - 1)$, $j = 1,\ldots,N_p$, where $x_1 = 75\,\mu\text{m}$, $x_{N_p} = 1875\,\mu\text{m}$, and $N_p = 100$. We then fit a linear spline over the average cell density data at each point x at $t = 0$ from the data from Jin et al. [1] mentioned, and evaluate this spline at each x_j, $j = 1,\ldots,N_p$. These splines are fit using the implementation of the Dierckx Fortran library [2] in Julia available from the `Dierckx` package [3]. We then prescribe $T(t;\boldsymbol{\alpha})$, $D(u;\boldsymbol{\beta})$, and $R(u;\boldsymbol{\gamma})$, along with the respective parameters for each function, and solve the corresponding partial differential equation (PDE). We store the solutions at $t = 0, 12, 24, 36, 48\,\text{h}$, giving solutions $\widehat{u}_{ij}$ for the density at $x = x_i$ and $t = t_j$, with $x_i = x_1 + (i-1)\Delta x$ and $t_j = (j-1)\Delta t$, $\Delta t = 12$, for $i = 1,\ldots,N_p$ and $j = 1,\ldots,5$. To then add noise to this data, we

replace each $\widehat{u}_{ij}$ with $\widehat{u}_{ij} + |\widehat{u}_{ij}|^{0.45} z_{ij}$, where z_{ij} is a sample from $\mathcal{N}(0, 1)$; to ensure the densities remain positive, any negative values from this replacement are further replaced by zero. Finally, to induce sparsity in the data, we thin the data randomly so that only 190 points remain, with 38 points at each time t_j, $j = 1, \ldots, 5$.

All results in these studies are presented in the same units as in Jin et al. [1], but in our implementation we put space and time into units of mm and d, respectively.

S1.1 Study I: Fisher-Kolmogorov model without delay

The first study we consider is a model of the form

$$\frac{\partial u}{\partial t} = \beta_1 \frac{\partial^2 u}{\partial x^2} + \gamma_1 u \left(1 - \frac{u}{K}\right), \tag{B}$$

where $K = 1.7 \times 10^{-3}\,\text{cells}\,\mu\text{m}^{-2}$. We use $\beta_1 = 301\,\mu\text{m}^2\,\text{h}^{-1}$ and $\gamma_1 = 0.044\,\text{h}^{-1}$. The data simulated from this model, along with a Gaussian process fit to the data, are shown in Fig A.

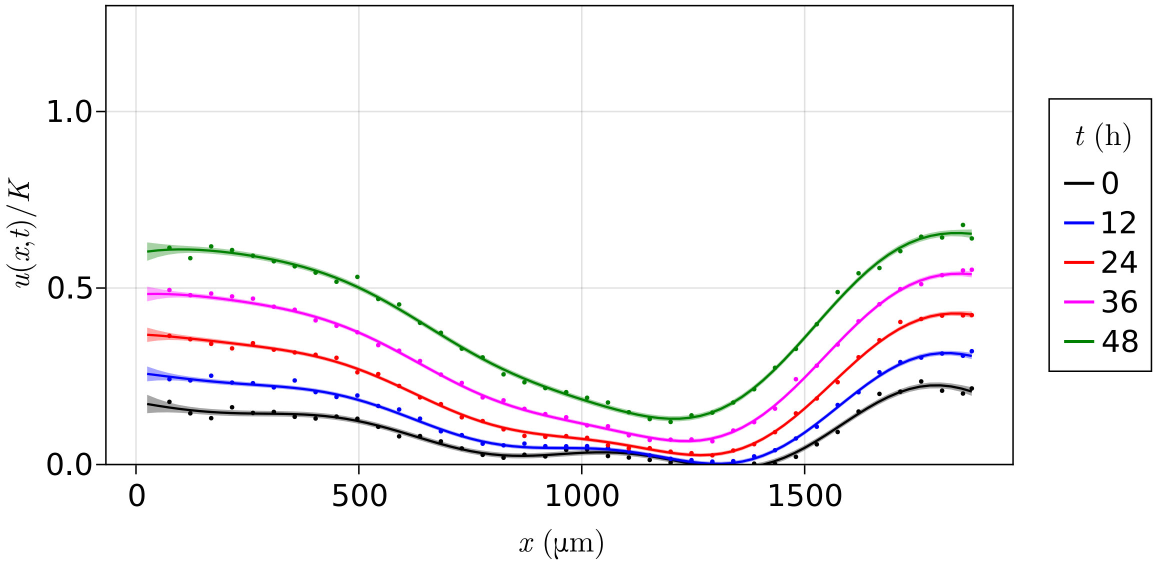

Figure A: Simulated data and fitted Gaussian process. Simulated data according to the model (B) with $D(u; \boldsymbol{\beta}) = \beta_1$ and $R(u; \boldsymbol{\gamma}) = \gamma_1 u(1 - u/K)$, with $\beta_1 = 301\,\mu\text{m}^2\,\text{h}^{-1}$ and $\gamma_1 = 0.044\,\text{h}^{-1}$. The lines and shaded regions represent a Gaussian process fit to the spatiotemporal data.

To begin with this model, we start with assuming that $T(t; \boldsymbol{\alpha}) = 1$, $D(u; \boldsymbol{\beta}) = \beta_1 + \beta_2(u/K)$, and $R(u; \boldsymbol{\gamma}) = \gamma_1 u(1 - u/K)$. This nonlinear diffusivity function is misspecified, with the true function corresponding to $\beta_2 = 0$. With this model we start with 10 bootstrap iterations and four optimisation restarts, sampling $\beta_1 \in [250, 1000]$, $\beta_2 \in [250, 1000]$, and $\gamma_1 \in [0.035, 0.05]$; note that $[\beta_1] = \mu\text{m}^2\,\text{h}^{-1}$,

$[\beta_2] = \mu m^2\,h^{-1}$, and $[\gamma_1] = h^{-1}$, where $[y]$ denotes the units of y. The results from this procedure are shown in Fig B. We see that we nearly capture the value of β_1 at a 95% level, although β_2 is not zero.

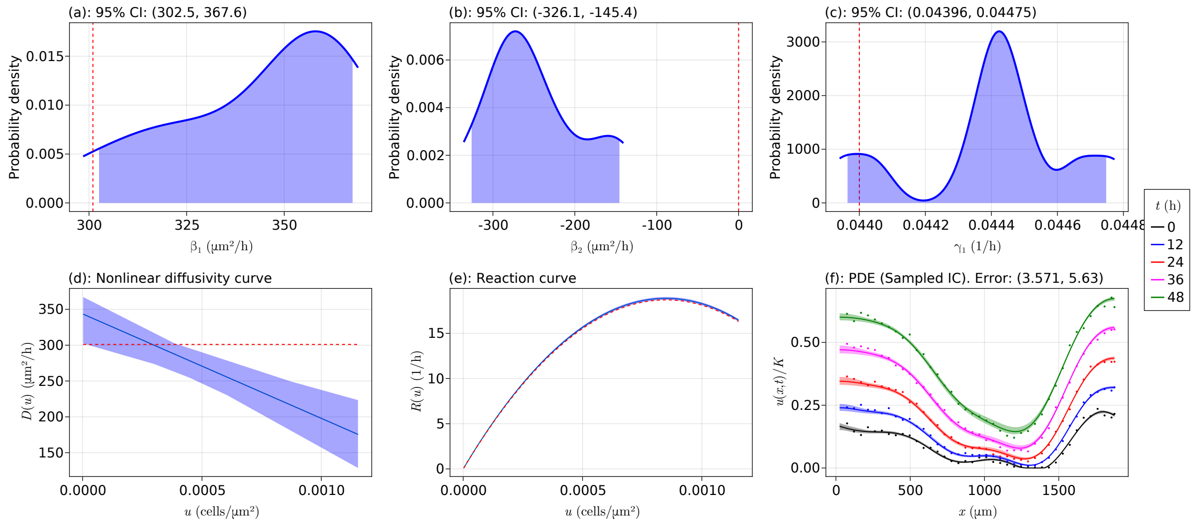

Figure B: Initial simulation results. Initial simulation results for the data from model (B) where $R(u;\boldsymbol{\gamma}) = \gamma_1 u(1-u/K)$ and we use the misspecified nonlinear diffusivity model $D(u;\boldsymbol{\gamma}) = \beta_1 + \beta_2(u/K)$. In the density plots, the dashed red vertical line is at the position of the true parameter value, and in the curve plots the dashed red curve shows the true mechanisms.

To go further, and to make the optimisation more efficient, we rescale our problem so that

$$D(u;\boldsymbol{\beta}) = 300\widehat{\beta}_1 - 200\widehat{\beta}_2\left(\frac{u}{K}\right) \quad \text{and} \quad R(u;\boldsymbol{\gamma}) = 0.044\widehat{\gamma}_1 u\left(1-\frac{u}{K}\right). \tag{C}$$

This puts all the parameters on the same scale. With this rescaling we perform $B = 200$ bootstrap iterations and four optimisation restarts around parameter values of 1. The revised results after rescaling are given in Fig C. We see that we have captured the true values of β_1 and γ_1, although β_2 is given by the uncertainty interval $(-326.1, -145.4)\ \mu m^2\,h^{-1}$ rather than its true value of $0\,\mu m^2\,h^{-1}$. We note that the uncertainty around the nonlinear diffusivity curve in (d) is correct for small densities, and most of the data are around these small values as shown in Fig A. Thus, the mechanism is still correct for small time, and it is not of concern that we have a different mechanism for larger densities given that there is only little data in this region.

Now let us obtain these results when we instead correctly specify $D(u;\boldsymbol{\beta}) = \beta_1$. We start with 10 bootstrap iterations and four optimisation restarts as before, sampling $\beta_1 \in [291.667, 541.6667]$ and $\gamma_1 \in [0.0380, 0.0420]$. The initial results are shown in Fig D, where we have captured γ_1 but only just missed the value of β_1.

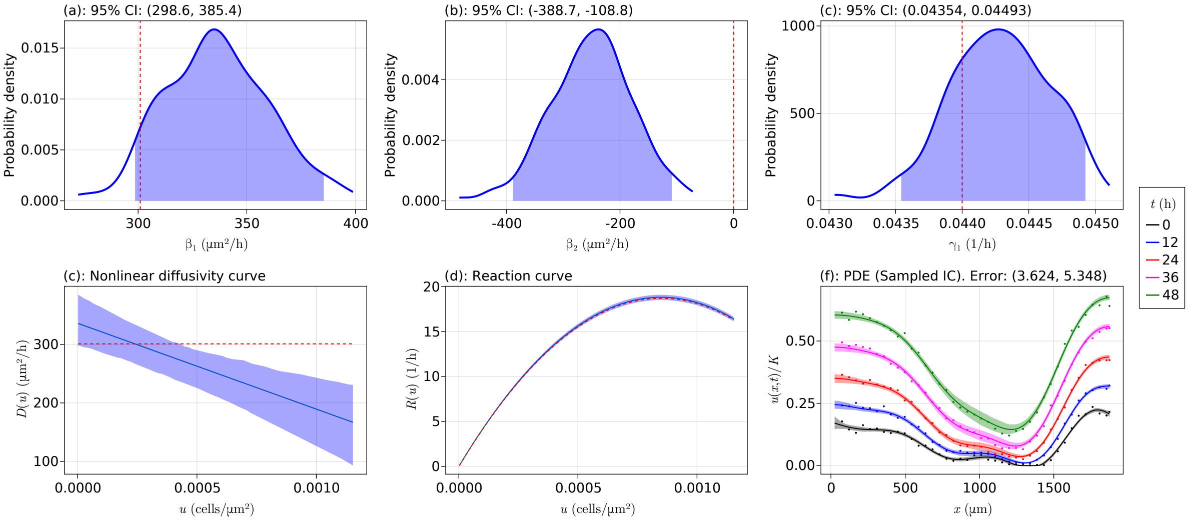

Figure C: Final simulation results. Final simulation results after re-scaling for the data from model (B) where $R(u;\boldsymbol{\gamma}) = \gamma_1 u(1-u/K)$ and we use the misspecified nonlinear diffusivity model $D(u;\boldsymbol{\gamma}) = \beta_1 + \beta_2(u/K)$. The re-scaling used is given in (C). In the density plots, the dashed red vertical line is at the position of the true parameter value, and in the curve plots the dashed red curve shows the true mechanisms.

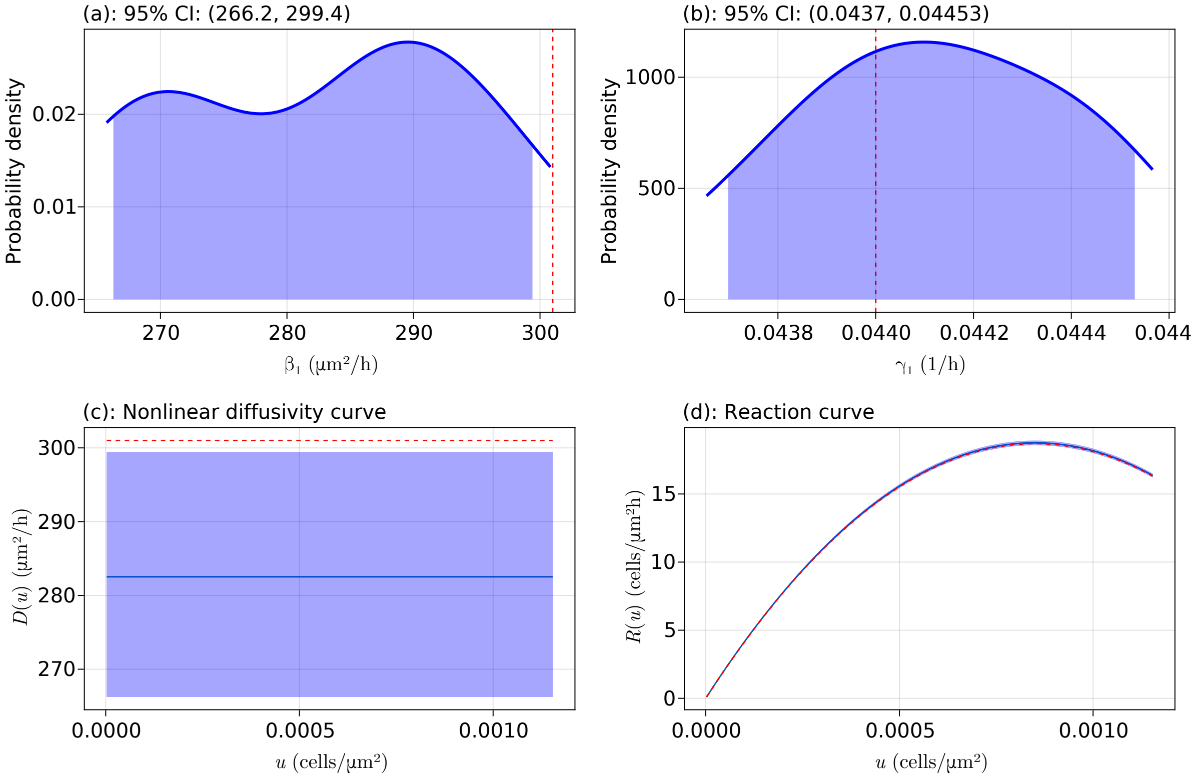

Figure D: Initial simulation results. Initial simulation results for the data from model (B) where $R(u;\boldsymbol{\gamma}) = \gamma_1 u(1-u/K)$ and we use the correctly specified model $D(u;\boldsymbol{\gamma}) = \beta_1$. In the density plots, the dashed red vertical line is at the position of the true parameter value, and in the curve plots the dashed red curve shows the true mechanisms.

The results in Fig D suggest the rescaling

$$D(u;\boldsymbol{\beta}) = 300\widehat{\beta}_1 \quad \text{and} \quad R(u;\boldsymbol{\gamma}) = 0.044\widehat{\gamma}_1 u\left(1 - \frac{u}{K}\right). \tag{D}$$

We perform 200 bootstrap iterations with this rescaled model and use four optimisation restarts. The final results are given in Fig E, where we now see that we have captured the true functional forms.

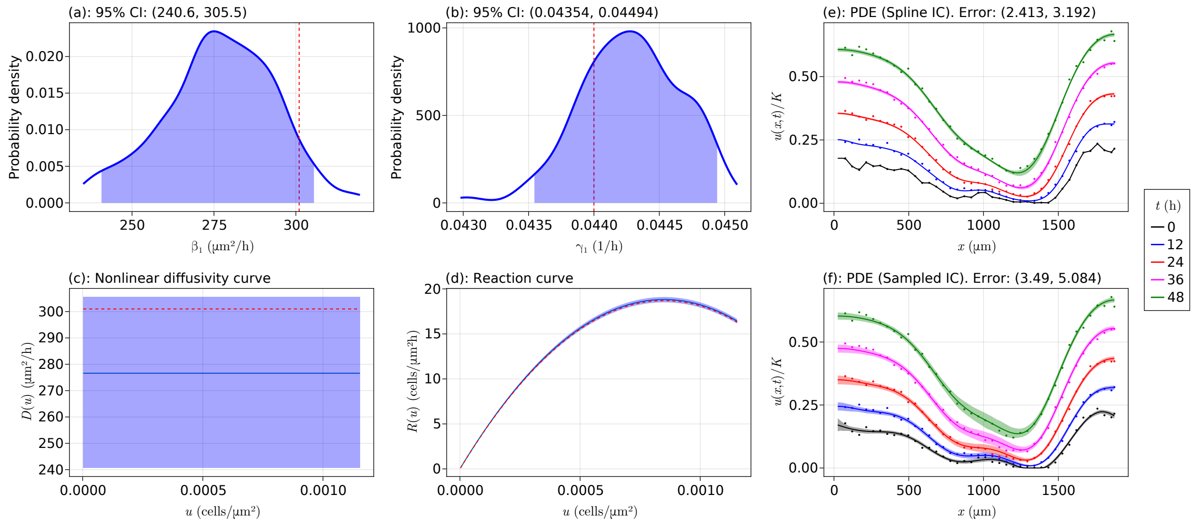

Figure E: Final simulation results. Final simulation results after re-scaling for the data from model (B) where $R(u;\boldsymbol{\gamma}) = \gamma_1 u(1-u/K)$ and we use the correctly specified nonlinear diffusivity model $D(u;\boldsymbol{\gamma}) = \beta_1$. The rescaling used is $\beta_1 = 300\widehat{\beta}_1$ and $\gamma_1 = 0.044\widehat{\gamma}_1$. In the density plots, the dashed red vertical line is at the position of the true parameter value, and in the curve plots the dashed red curve shows the true mechanism.

Let us now use our model selection procedure for comparing the two models in Figs C and E. These results are shown in Table A. We see that the β_1 estimates are reasonably similar, and the γ_1 estimates are the same across each model. The probability that the models are in the class of optimal models for the data, $\mathbb{P}(E_1)$, is 6.0% for the $D(u;\boldsymbol{\gamma}) = \beta_1 + \beta_2(u/K)$ model and 98.5% for the $D(u;\boldsymbol{\beta}) = \beta_1$ model. In particular, our model selection procedure has correctly identified the correct model out of the two models 98.5% of the time.

	$D(u;\boldsymbol{\beta})$	
	$\beta_1 + \beta_2\left(\frac{u}{K}\right)$	β_1
PDE Error (%)	$(3.624, 5.348)$	$(3.49, 5.084)$
β_1 ($\mu\mathrm{m}^2\,\mathrm{h}^{-1}$)	$(298.591, 385.381)$	$(240.633, 305.532)$
β_2 ($\mu\mathrm{m}^2\,\mathrm{h}^{-1}$)	$(-388.67, -108.825)$	—
γ_1 (h^{-1})	$(0.044, 0.045)$	$(0.044, 0.045)$
$\mathbb{P}(E_1)$	0.060	0.985
$\mathbb{P}(E_2)$	0.065	0.005
$\mathbb{P}(E_3)$	0.875	0.010

Table A: Results for the two models considered. Interval estimates, PDE errors, and model selection results for the two nonlinear diffusivity functions considered in this study. The PDE errors are computed using sampled initial conditions. Significance levels for all uncertainty intervals are 95%. For both models we use $R(u;\boldsymbol{\gamma}) = \gamma_1 u(1-u/K)$ and $T(t;\boldsymbol{\alpha}) = 1$.

S1.2 Study II: Fisher-Kolmogorov model with delay

We now consider simulating data from the model

$$\frac{\partial u}{\partial t} = \frac{1}{1+\exp(-\alpha_1 - \alpha_2 t)}\left[\beta_1 \frac{\partial^2 u}{\partial x^2} + \gamma_1 u\left(1 - \frac{u}{K}\right)\right], \tag{E}$$

where $\alpha_1 = -1.50$, $\alpha_2 = 0.31\,\mathrm{h}^{-1}$, $\beta_1 = 571\,\mu\mathrm{m}^2\,\mathrm{h}^{-1}$, and $\gamma_1 = 0.081\,\mathrm{h}^{-1}$. The data simulated from this model, along with a Gaussian process fit to the data, are shown in Fig F.

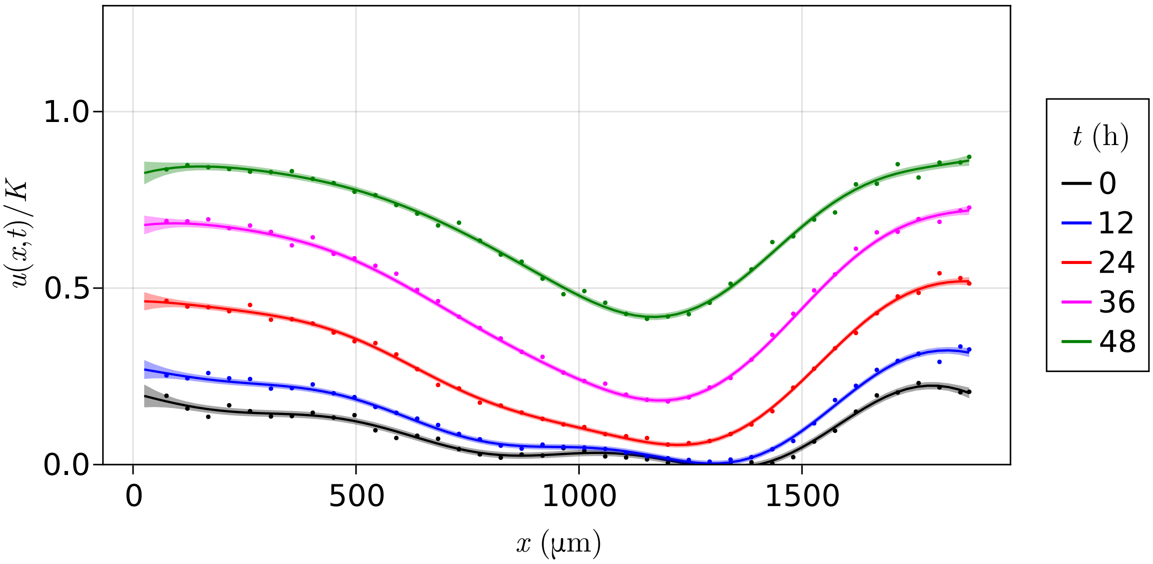

Figure F: Simulated data and fitted Gaussian process. Simulated data according to the model (E) with $\alpha_1 = -1.5$, $\alpha_2 = 0.31\,\mathrm{h}^{-1}$, $\beta_1 = 571\,\mu\mathrm{m}^2\,\mathrm{h}^{-1}$, and $\gamma_1 = 0.081\,\mathrm{h}^{-1}$. The lines and shaded regions represent a Gaussian process fit to the spatiotemporal data.

We consider fitting the following five models to this data:

$$\text{Model 1}: \ T(t;\boldsymbol{\alpha}) = 1, \qquad D(u;\boldsymbol{\beta}) = \beta_1, \tag{F}$$

$$\text{Model 2}: \ T(t;\boldsymbol{\alpha}) = \frac{1}{1+\exp(-\alpha_1 - \alpha_2 t)}, \quad D(u;\boldsymbol{\beta}) = \beta_1, \tag{G}$$

$$\text{Model 3}: \ T(t;\boldsymbol{\alpha}) = 1, \qquad D(u;\boldsymbol{\beta}) = \beta_2\left(\frac{u}{K}\right), \tag{H}$$

$$\text{Model 4}: \ T(t;\boldsymbol{\alpha}) = \frac{1}{1+\exp(-\alpha_1 - \alpha_2 t)}, \quad D(u;\boldsymbol{\beta}) = \beta_2\left(\frac{u}{K}\right), \tag{I}$$

$$\text{Model 5}: \ T(t;\boldsymbol{\alpha}) = \frac{1}{1+\exp(-\alpha_1 - \alpha_2 t)}, \quad D(u;\boldsymbol{\beta}) = \beta_1 + \beta_2\left(\frac{u}{K}\right)^{\beta_3}, \tag{J}$$

with $R(u;\boldsymbol{\gamma}) = \gamma_1 u(1 - u/K)$ for each model. We note that these are the same five models considered in the main body of the manuscript. We do not show the preliminary results used to obtain the scaling for the parameters in each of these models as we did in the previous study, but note that the procedure

remains the same. For each model, after rescaling, we use 100 bootstrap iterations with no optimiser restarts, starting each parameter at 1. The final results are shown in Table B.

	Model				
	Model 1	Model 2	Model 3	Model 4	Model 5
PDE Error (%)	$(7.978, 10.730)$	$(2.600, 4.556)$	$(8.731, 11.598)$	$(3.864, 6.312)$	$(2.605, 4.329)$
α_1	—	$(-1.723, -0.583)$	—	$(-1.599, -0.441)$	$(-1.843, -0.540)$
α_2 (h^{-1})	—	$(0.172, 0.326)$	—	$(0.162, 0.330)$	$(0.169, 0.345)$
β_1 ($\mu\mathrm{m}^2\,\mathrm{h}^{-1}$)	$(424.904, 567.49)$	$(483.627, 620.185)$	—	—	$(316.126, 407.045)$
β_2 ($\mu\mathrm{m}^2\,\mathrm{h}^{-1}$)	—	—	$(1703.240, 2253.560)$	$(1547.660, 2281.510)$	$(130.914, 424.938)$
β_3	—	—	—	—	$(0.128, 0.457)$
γ_1 (h^{-1})	$(0.068, 0.071)$	$(0.080, 0.083)$	$(0.069, 0.072)$	$(0.079, 0.084)$	$(0.080, 0.083)$
$\mathbb{P}(E_1)$	0.000	0.660	0.000	0.000	0.560
$\mathbb{P}(E_2)$	0.000	0.160	0.000	0.000	0.180
$\mathbb{P}(E_3)$	1.000	0.180	1.000	1.000	0.260

Table B: Results for the five models considered. Interval estimates, PDE errors, and model selection results for the five models (F)–(J) considered for the data generated by (E). 100 bootstrap iterations are used for each model. The PDE errors are computed using sampled initial conditions. Significance levels for all uncertainty intervals are 95%.

See that for the model in Table B which is correctly specified, (G), the correct parameter values are captured within the uncertainty intervals for the respective parameters. When we do not include delay but give the correct cell migration mechanism, (F), we nearly recover the correct diffusivity β_1 but no longer obtain the correct reaction coefficient γ_1, leading to a significant increase in the PDE error. For the Porous-Fisher model with delay, (I), we see that the delay and reaction mechanisms are recovered, but the error is still slightly larger than for (G). The error is significantly increased in (H) when we do not include delay. Finally, the generalised Porous-FKPP model (J) recovers the correct delay and reaction terms. This model also gives an error that is very similar to (G). Finally, in terms of model selection, we notice that all the models without delay and the Porous-Fisher model with delay are never selected as falling within the class of optimal models for this data. The correctly specified model, (G), is correctly selected 66% of the time, with the next best model being the generalised Porous-FKPP model (J). We show in Fig G the results for the correctly specified model in (G), where we can indeed see that the model fits the data well and all the mechanisms are recovered.

To further understand how model misspecification can present itself from the results, in Fig H we plot the PDE solutions for all the models in Table B. In (a), where only the delay term is missing, we see that the curves are initially over the data, but eventually fall below it — this feature implies the need for delay. In (c), which misspecifies both the delay and the diffusion, we again see the same type of delay where the curves start above and go below. In addition, we see that the cell densities are clearly problematic, namely our model fails to predict how the cells move into the centre of the well. That is, the diffusion term is misspecified. Hence, an incorrect diffusion term can be seen through a poor migration of cell densities compared to the experimental data. This feature is also present in (d), except here in (d) the delay term is correctly specified and so we do not see the curves going below and then over. Finally,

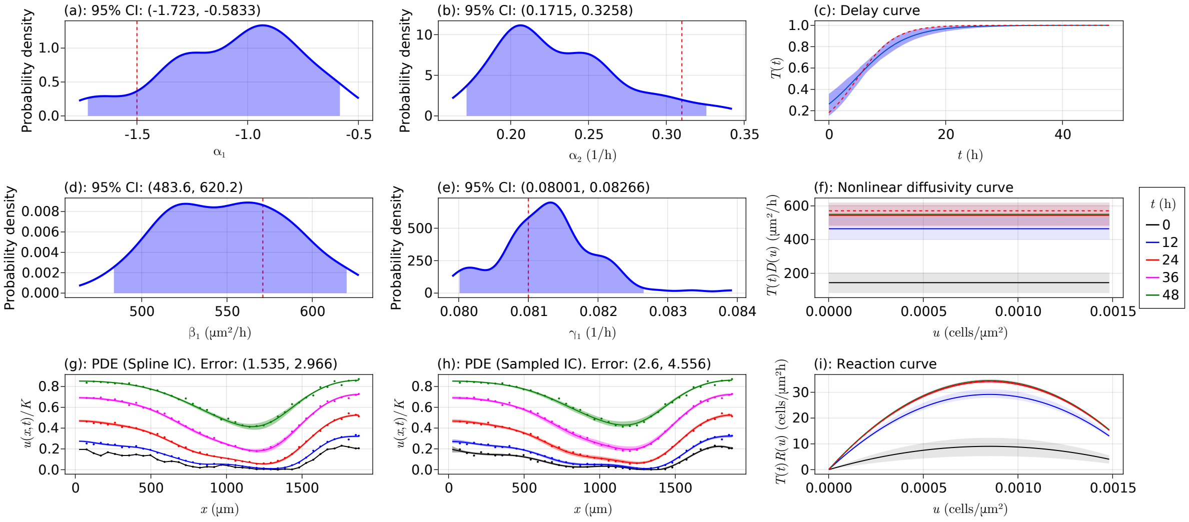

Figure G: Final model results for the optimal model. Results for the fitted model in (G) for the data generated from (E). In the density plots, the dashed red vertical line is at the position of the true parameter value, in the curve plots the dashed red curve shows the true mechanism, and in the curve plots the different shaded regions show various values of the products of the respective mechanisms at the shown times.

in (e), where the delay term is specified and the correct model is seen only where $\beta_2 = 0$, it is hard to see any reason for the model to be incorrect, and indeed that is what we learned from Table B. The way to work with this model in practice may be to first try a simpler model (which would be the correct model in this instance) and the more complex model, and comparing them directly with our probability estimates. In summary, we have the following:

1. A missing delay term can be identified by seeing that the learned curves start below (resp. above) the data and then, as time increases, go above (resp. below) the data.
2. A misspecified diffusion term can be identified by inferring from the cell densities the migration behaviour of the cells compared to the data. In our case, we saw that the cells were predicted to migrate into the middle of the well in a way that was not like what we see from the data, thus indicating an incorrect diffusion term.

S2 Basis function approach

In this section we present an alternative to the nonlinear optimisation problem in (35) for problems without delay and whose nonlinear diffusivity and reaction functions can be represented as linear com-

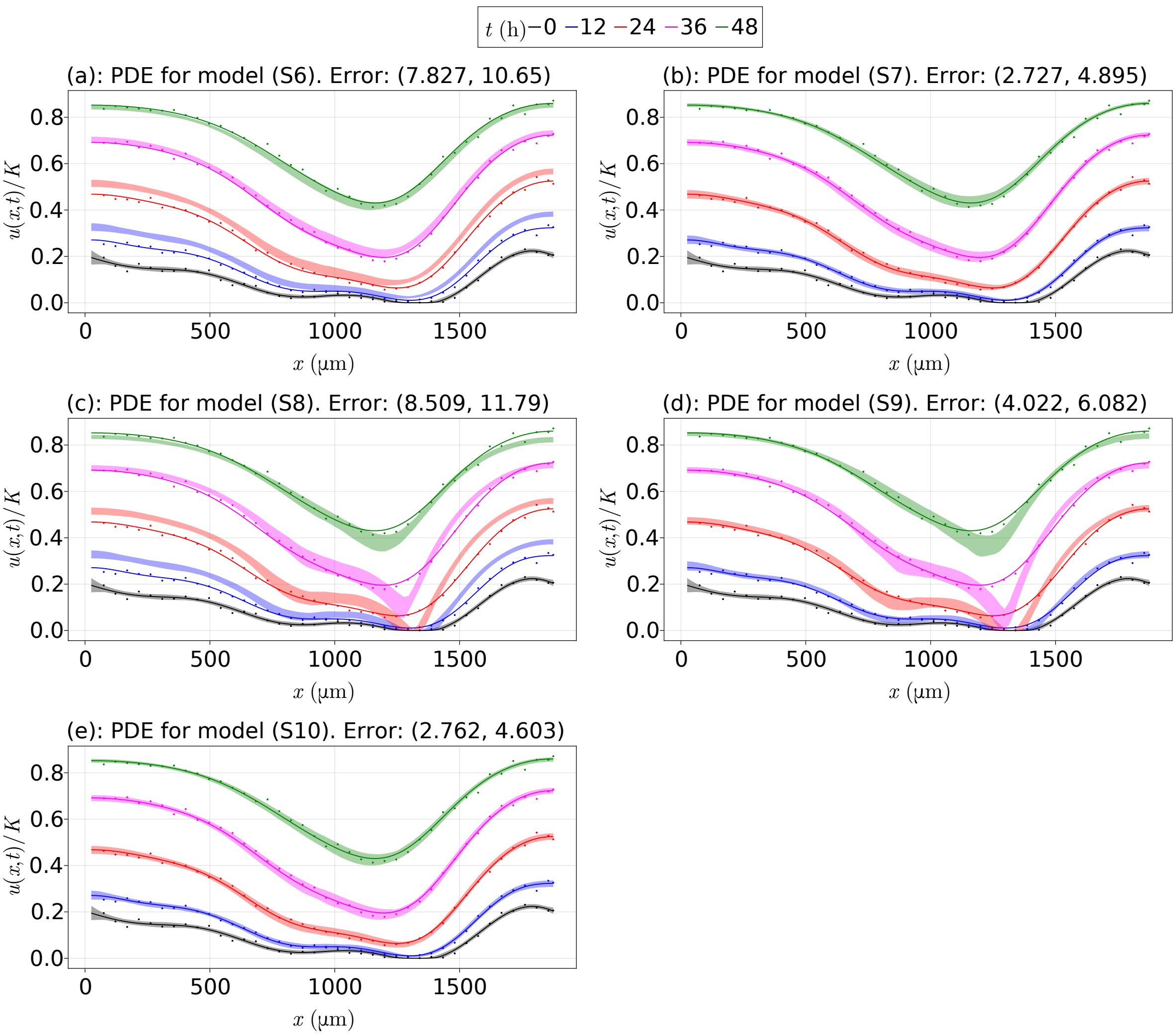

Figure H: All model results for the tested models. All PDE solutions for the fitted models in Table B. The initial conditions are sampled using the Gaussian process. The correctly specified model's results are those in (b).

binations of individual basis functions,

$$D(u;\boldsymbol{\beta}) = \sum_{k=1}^{d} \beta_k \phi_k(u) \quad \text{and} \quad R(u;\boldsymbol{\gamma}) = \sum_{k=1}^{r} \gamma_k \psi_k(u), \tag{K}$$

where the basis functions ϕ_k and ψ_k, for each $k = 1, \ldots, d$ and $k = 1, \ldots, r$, respectively, are differentiable. With these functions, (32) becomes

$$\frac{\partial u_{ij}^*}{\partial t} = \sum_{k=1}^{d} \left[\phi_k'(u_{ij}^*)\left(\frac{\partial u_{ij}^*}{\partial x}\right)^2 + \phi_k(u_{ij}^*)\frac{\partial^2 u_{ij}^*}{\partial x^2}\right]\beta_k + \sum_{k=1}^{r}\gamma_k\psi_k(u_{ij}^*), \tag{L}$$

where primes denote derivatives in u. We then define the matrices $\mathbf{A}_1 \in \mathbb{R}^{nm\times d}$ and $\mathbf{A}_2 \in \mathbb{R}^{nm\times r}$ such that

$$[\mathbf{A}_1]_{(i,j),k} = \phi_k(u_{ij}^*)\frac{\partial^2 u_{ij}^*}{\partial x^2} + \phi_k'(u_{ij}^*)\left(\frac{\partial u_{ij}^*}{\partial x}\right)^2 \quad \text{and} \quad [\mathbf{A}_2]_{(i,j),k} = \psi_k(u_{ij}^*), \tag{M}$$

respectively, where $[\mathbf{A}_\ell]_{(i,j),k}$ denotes the entry in $\mathbf{A}_\ell$ in the kth column in the row corresponding to the point (ξ_i, τ_j) for $i = 1, \ldots, n$ and $j = 1, \ldots, m$, and $\ell = 1, 2$. Next, define $\mathbf{A} = [\mathbf{A}_1\ \mathbf{A}_2] \in \mathbb{R}^{nm \times (d+r)}$ and $\boldsymbol{\theta} = (\boldsymbol{\beta}^\mathsf{T}, \boldsymbol{\gamma}^\mathsf{T})^\mathsf{T}$. Given this form we obtain the matrix system

$$\mathbf{A}\boldsymbol{\theta} = \frac{\partial \mathbf{u}^*}{\partial x}. \tag{N}$$

Equation (N) can then be solved for $\boldsymbol{\theta}$ using the backslash operator in Julia [4]. This approach could be especially useful for obtaining initial scale estimates for parameters, for example, rather than dealing with the far slower nonlinear optimisation problem on an unscaled problem to start with.

We note that while this approach could be suitable for say a Porous-Fisher PDE,

$$\frac{\partial u}{\partial t} = \beta_1 \frac{\partial}{\partial x}\left[\left(\frac{u}{K}\right)\frac{\partial u}{\partial x}\right] + \gamma_1 u\left(1 - \frac{u}{K}\right), \tag{O}$$

it would not be suitable if there were any nonlinear delay or if we had for example the PDE

$$\frac{\partial u}{\partial t} = \frac{\partial}{\partial x}\left[\beta_1 \mathrm{e}^{-\beta_2 u}\left(\frac{u}{K}\right)\frac{\partial u}{\partial x}\right] + \gamma_1 u\left(1 - \frac{u}{K}\right), \tag{P}$$

since the nonlinear diffusivity function cannot be represented as a linear combination of basis functions. We could still approximate this problem such that the functions are linear in the parameters, for example by temporarily fixing β_2 at a value, say $\beta_2 = 1$, and solving (N) to estimate β_1. We could then fix β_1 at this found value and estimate $\mathrm{e}^{-\beta_2 u} \approx 1 - \beta_2 u$, assuming $\beta_2 u$ is small, and estimate β_2 in the same way. This procedure of fitting β_1 and then β_2 separately could be repeated if desired. These approximations would give rough scales of the estimates that could be used to rescale the parameters and make the nonlinear optimisation problem in (35) significantly faster.

Let us compare the results from this approach to those given in Section S1.1, writing

$$D(u; \boldsymbol{\beta}) = \beta_1 \phi_1(u) \quad \text{and} \quad R(u; \boldsymbol{\gamma}) = \gamma_1 \psi_1(u), \tag{Q}$$

where $\phi_1(u) \equiv 1$ and $\psi_1(u) \equiv u(1 - u/K)$. The final results are shown in Fig I with 200 bootstrap iterations. The results are very similar to those in Fig E.

We note that these similarities make sense if we recall that the solution to (N) is equivalent to solving

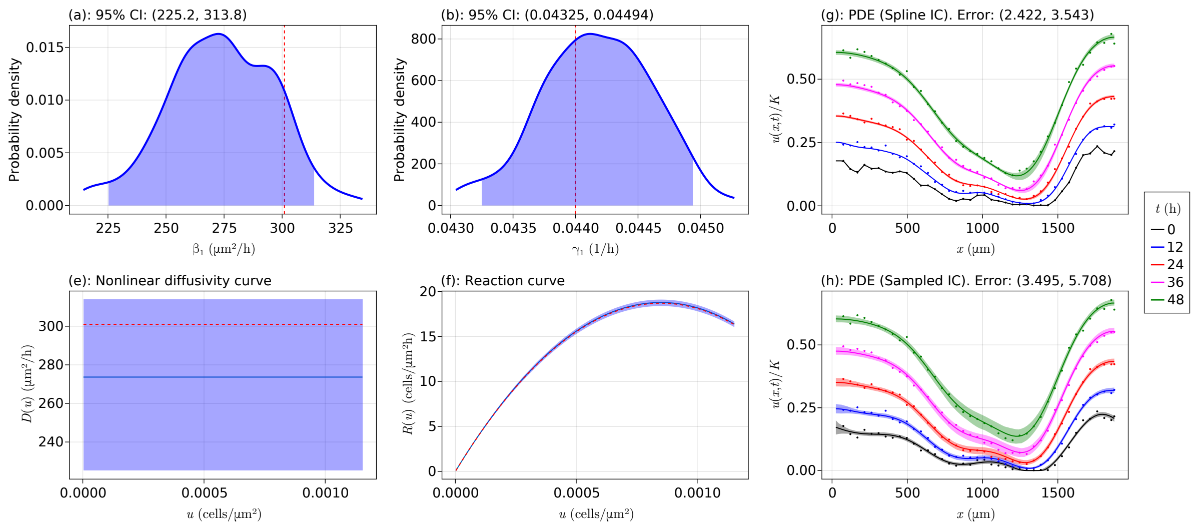

Figure I: Results for the Fisher-Kolmogorov model without delay using the basis function approach. Results for parameters, functional forms, and PDE solutions for the model equation (B) using the basis function approach. In the density plots, the dashed red line is at the true parameter value, and in the curve plots the dashed red curve shows the true curve.

the least squares problem

$$\widehat{\boldsymbol{\theta}} = \operatorname*{argmin}_{\boldsymbol{\theta}\in\mathbb{R}^{d+r}} \mathcal{L}(\boldsymbol{\theta}), \tag{R}$$

$$\mathcal{L}(\boldsymbol{\theta}) = \sum_{I=1}^{n}\sum_{j=1}^{m}\left\{\frac{\partial u_{ij}^*}{\partial t} - \left(\sum_{k=1}^{d}\left[\phi_k'(u_{ij}^*)\left(\frac{\partial u_{ij}^*}{\partial x}\right)^2 + \phi_k(u_{ij}^*)\frac{\partial^2 u_{ij}^*}{\partial x^2}\right]\beta_k + \sum_{k=1}^{r}\gamma_k\psi_k(u_{ij}^*)\right)\right\}^2, \tag{S}$$

where the summand is the difference between the terms on both sides of (L). See that this optimisatiom problem is the same as minimising only $\mathcal{L}_{\text{PDE}}$ in (33) and not considering $\mathcal{L}_{\text{GLS}}$ in (34).

We note that for more complicated diffusivity models, such as the quadratic model $D(u;\boldsymbol{\beta}) = \beta_1 + \beta_2(u/K) + \beta_3(u/K)^2$, we find results with increased PDE errors despite similarly learned mechanisms relative to the nonlinear least squares approach. We explain this by noting that, in this approach, the parameter estimation is focusing only on our sampled values without any direct consideration for the actual data as would be accomplished through $\mathcal{L}_{\text{GLS}}$. This failure to capture the data during the bootstrapping procedure using the basis function approach demonstrates one downside.

The results in this section allow us to make the following conclusions around this method. The approach is extremely fast relative to the main approach which requires solving (35). Nevertheless, the approach can lead to greater error in the PDE solutions due to not directly considering the data when estimating the parameters, depending on the complexity of the model. We therefore recommend this method as an efficient way to obtain scales for the parameters when the problem is linear, or for example as a quick way to see if delay may be needed in the problem. Once these scales are found, the coefficients

should be rescaled and the nonlinear optimisation problem in (35) should be used to give the final results. Future work could for example augment the loss function (S) with the loss function $\mathcal{L}_{\text{PDE}}$ or some other term that regularises the loss function in a way that considers the data in a more direct manner, although this may not lead to a simple matrix system as in (N).

S3 Results for the scratch assay data

In this section we present plots for a delayed Fisher-Kolmogorov model and a delayed generalised Porous-FKPP model, as presented in the main body of the manuscript, for each data set from Jin et al. [1]. We first show the final results for the delayed Fisher-Kolmogorov model and then we present those for the delayed generalised Porous-FKPP model.

S3.1 Delayed Fisher-Kolmogorov models

We start by presenting the plots for the delayed Fisher-Kolmogorov model, where $T(t; \boldsymbol{\alpha}) = 1/[1 + \exp(-\alpha_1 - \alpha_2 t)]$, $D(u; \boldsymbol{\beta}) = \beta_1$, and $R(u; \boldsymbol{\gamma}) = \gamma_1 u(1 - u/K)$, for each data set from Jin et al. [1]. The plots are shown in Figs J–O for the 10,000, 12,000, 14,000, 16,000, 18,000, and 20,000 CPW data sets, respectively. The density plots in (a), (b), (d), an (e) for each of these plots appear to be unimodal with well-defined peaks, implying that for these models the parameters have been well identified. For the delay curves in (c) of each plot, we see that the uncertainty around the curve is similar for each data set, although the uncertainty in Fig N(c) is a bit wider than the other plots and in Fig O(f) it is significantly smaller than the other plots. This issue in Fig O(f) is not surprising since we know in Fig O(h), and from the discussion in the manuscript, that there are some issues with this model for the 20,000 CPW data set. The reaction curves in (i) have very little uncertainty for large time, although for small time the uncertainty intervals are wider as the delay takes time to start. The opposite is true for the nonlinear diffusivity curves in (f), where we instead have little uncertainty initially, with increasing uncertainty as the cells start to proliferate at greater rates. For the PDE solutions, we see that while the PDE error is smaller in (g) of each plot where we use a spline initial condition, the uncertainty intervals do not typically match the data as well as they do in (h) where we use a sampled initial conditions. Overall, these results show good fits to the data and that we have identified the parameter coefficients reliably in each case, with the exception of the 20,000 CPW data set as discussed in the manuscript.

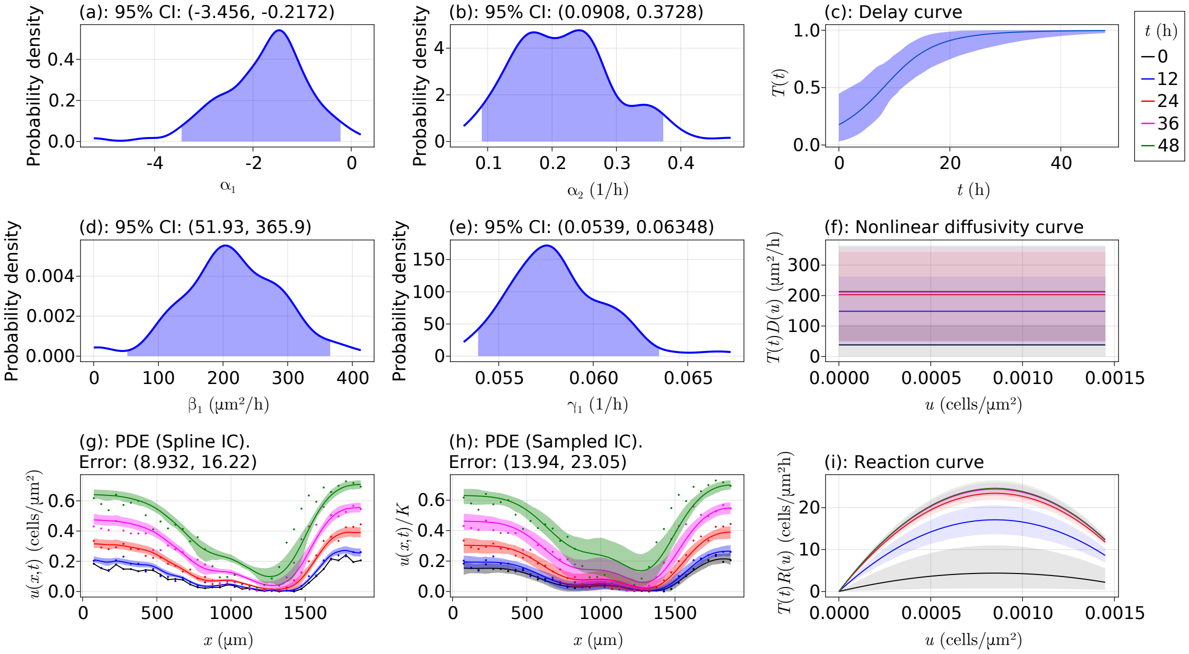

Figure J: Final model results for the optimal model with 10,000 cells per well. Results for the Fisher-Kolmogorov model with delay for the 10,000 cells per well data set from Table 1. In the curve plots, the different shaded regions show various values of the products of the respective mechanisms at the shown times. The shaded regions in the PDE plots represent the uncertainty at each point around the mean curve shown, and the points shown are the data from Jin et al. [1].

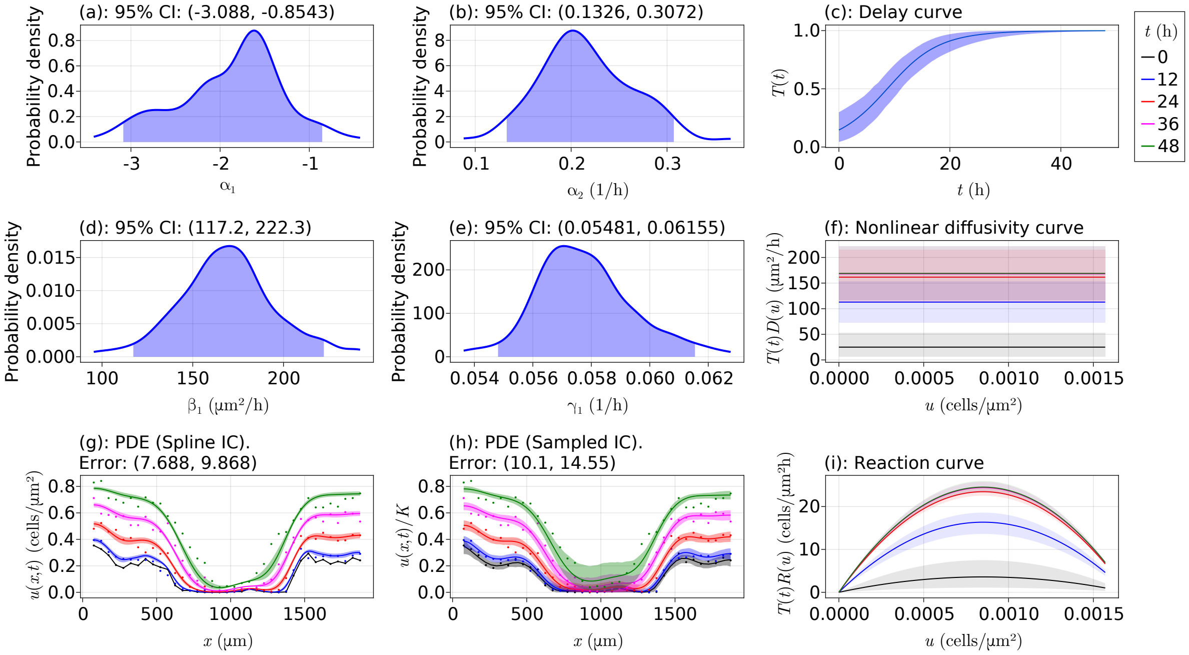

Figure K: Final model results for the optimal model with 12,000 cells per well. Results for the Fisher-Kolmogorov model with delay for the 12,000 cells per well data set from Table 1. In the curve plots, the different shaded regions show various values of the products of the respective mechanisms at the shown times. The shaded regions in the PDE plots represent the uncertainty at each point around the mean curve shown, and the points shown are the data from Jin et al. [1].

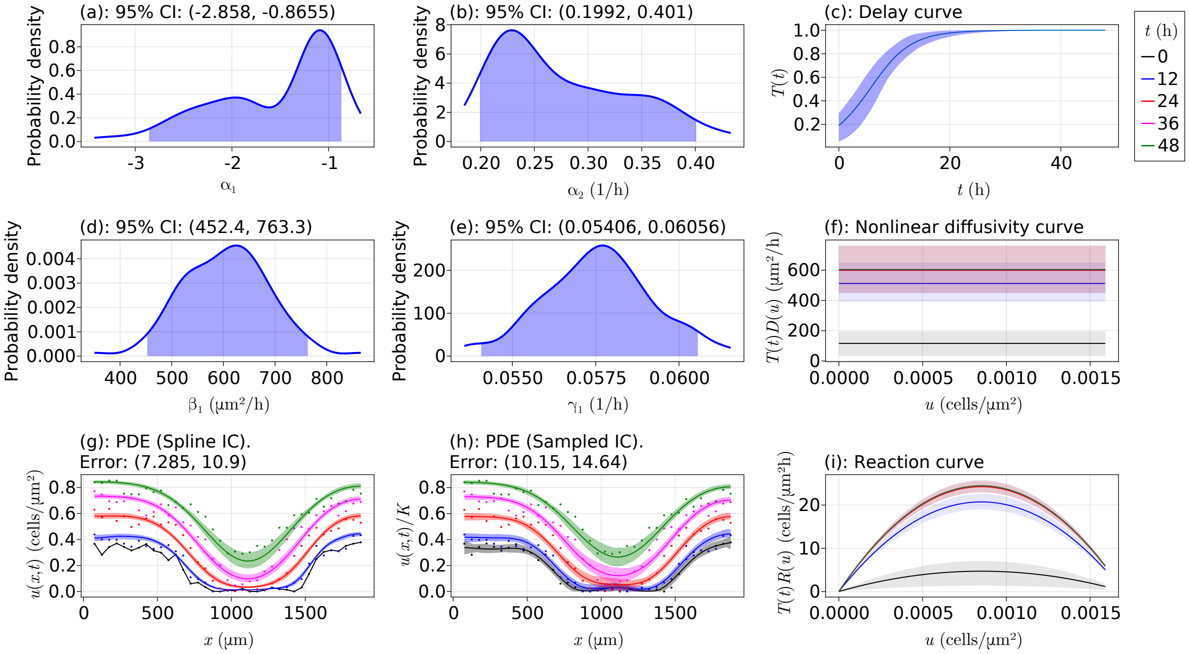

Figure L: Final model results for the optimal model with 14,000 cells per well. Results for the Fisher-Kolmogorov model with delay for the 14,000 cells per well data set from Table 1. In the curve plots, the different shaded regions show various values of the products of the respective mechanisms at the shown times. The shaded regions in the PDE plots represent the uncertainty at each point around the mean curve shown, and the points shown are the data from Jin et al. [1].

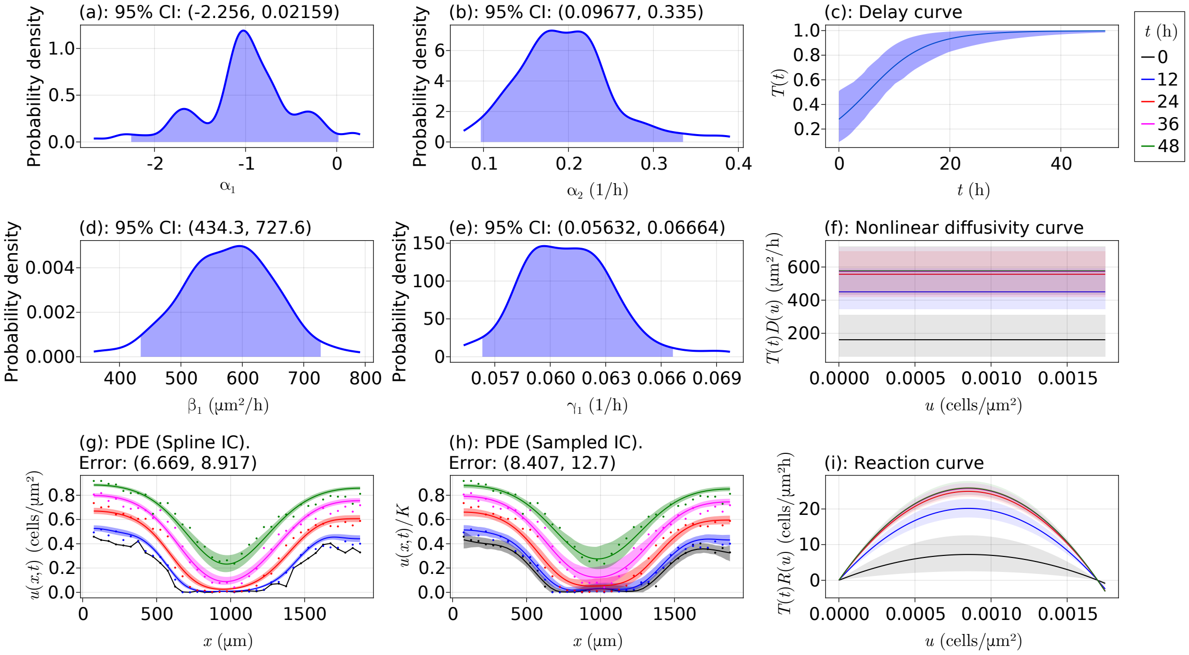

Figure M: Final model results for the optimal model with 16,000 cells per well. Results for the Fisher-Kolmogorov model with delay for the 16,000 cells per well data set from Table 1. In the curve plots, the different shaded regions show various values of the products of the respective mechanisms at the shown times. The shaded regions in the PDE plots represent the uncertainty at each point around the mean curve shown, and the points shown are the data from Jin et al. [1].

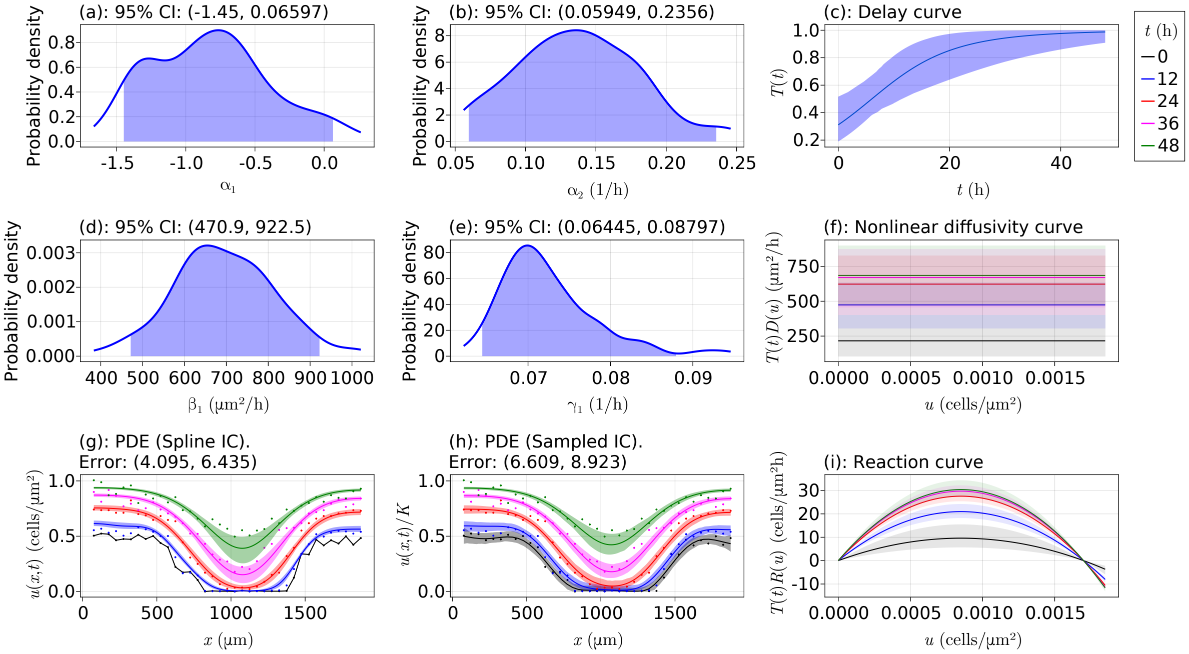

Figure N: Final model results for the optimal model with 18,000 cells per well. Results for the Fisher-Kolmogorov model with delay for the 18,000 cells per well data set from Table 1. In the curve plots, the different shaded regions show various values of the products of the respective mechanisms at the shown times. The shaded regions in the PDE plots represent the uncertainty at each point around the mean curve shown, and the points shown are the data from Jin et al. [1].

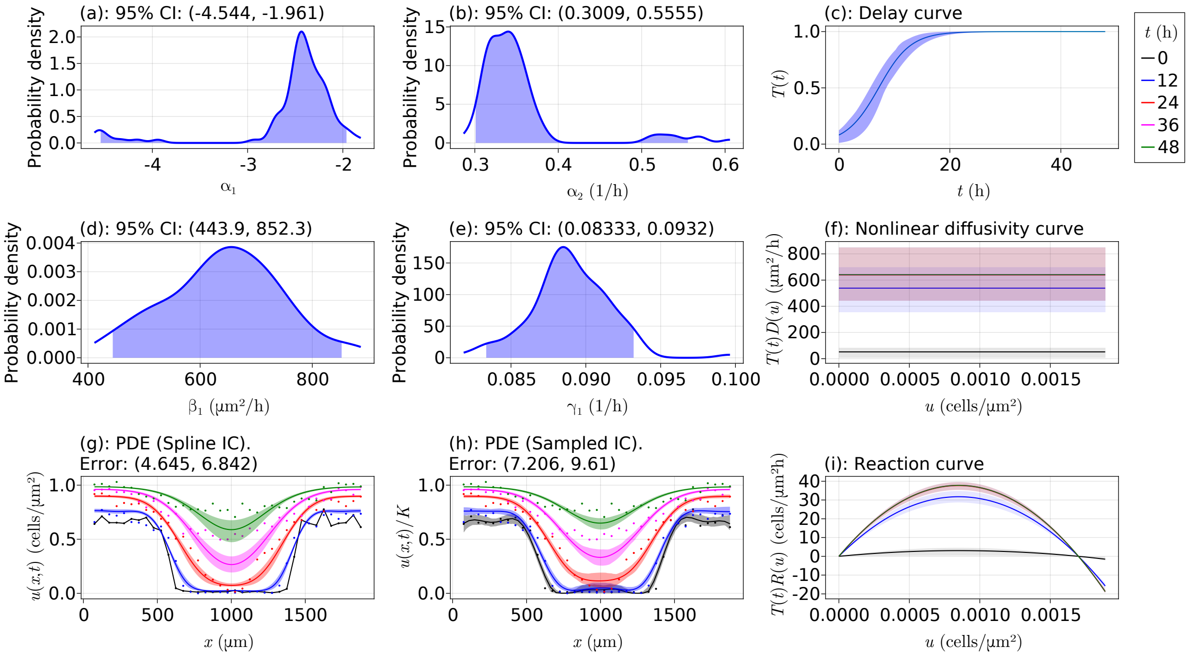

Figure O: Final model results for the optimal model with 20,000 cells per well. Results for the Fisher-Kolmogorov model with delay for the 20,000 cells per well data set from Table 1. In the curve plots, the different shaded regions show various values of the products of the respective mechanisms at the shown times. The shaded regions in the PDE plots represent the uncertainty at each point around the mean curve shown, and the points shown are the data from Jin et al. [1].

S3.2 Delayed Generalised Porous-FKPP models

In this section we show the results for the delayed generalised Porous-FKPP models, where $T(t;\boldsymbol{\alpha}) = 1/[1+\exp(-\alpha_1 - \alpha_2 t)]$, $D(u;\boldsymbol{\beta}) = \beta_1 + \beta_2(u/K)^{\beta_3}$, and $R(u;\boldsymbol{\gamma}) = \gamma_1 u(1-u/K)$, for each data set from Jin et al. [1]. This is the same model considered by Lagergren et al. [5]. The plots are shown in Figs P–U for the 10,000, 12,000, 14,000, 16,000, 18,000, and 20,000 CPW data sets, respectively. For these plots, the modes in the density plots in (a)–(f) of each plot are not as well-defined as they were for the delayed Fisher-Kolmogorov models, especially for β_1 and β_3, where the peak of the modes are typically very flat, showing issues with identifiability. Most of the plots are still unimodal. The behaviour of the uncertainty around the delay curves in (g) of each plot is similar to those for the delayed Fisher-Kolmogorov model. The nonlinear diffusivity curves in (h) of each plot show behaviour that is alike $D(u;\boldsymbol{\beta}) = \beta_1$ for smaller densities, especially for the 10,000 CPW data set. As the initial cell density increases, the range of values for u where the curve is completely flat is decreasing, where we see that the curves start to decrease sooner and the rate of decreasing increases with initial cell density, and in Fig U(h) we see that the nonlinear diffusivity curve is immediately decreasing. This observation supports the discussion in the manuscript where nutrients are running out for the cells when we increase the initial cell density, with greater cell densities leading to a greater need for these nutrients, or at least a different noise model for the Gaussian process to handle these differences. The reaction curves in (i) of each plot show no issues.

S4 Data thresholding

In this section we discuss a method for improving the quality of data used for estimating the parameters $\boldsymbol{\theta}$ by removing data points that violate certain conditions. We suppose we have some data $\mathbf{u}^*$ and corresponding derivatives $\partial\mathbf{u}^*/\partial t$, $\partial\mathbf{u}^*/\partial x$, and $\partial^2\mathbf{u}^*/\partial x^2$. We then define some threshold parameters $0 \leq \delta u, \delta t < 1$, where 0 corresponds to no thresholding and 1 means all points are removed. To motivate what conditions we should use to discard data, let us consider the space-time diagrams in Fig 5. We see that there are some regions where there are very little cells so that the density is almost zero, and there are some regions where the density evolves very slowly in time. These values would pollute the larger sum in (33), and so it would be useful to remove them as they do not contribute any valuable information. Thus, we define the following three conditions that must be satisfied for a data point to be used, where u_{ij}^* is an estimate for the cell density from the Gaussian process at (ξ_i, τ_j) for $i = 1, \ldots, n$ and $j = 1, \ldots, m$:

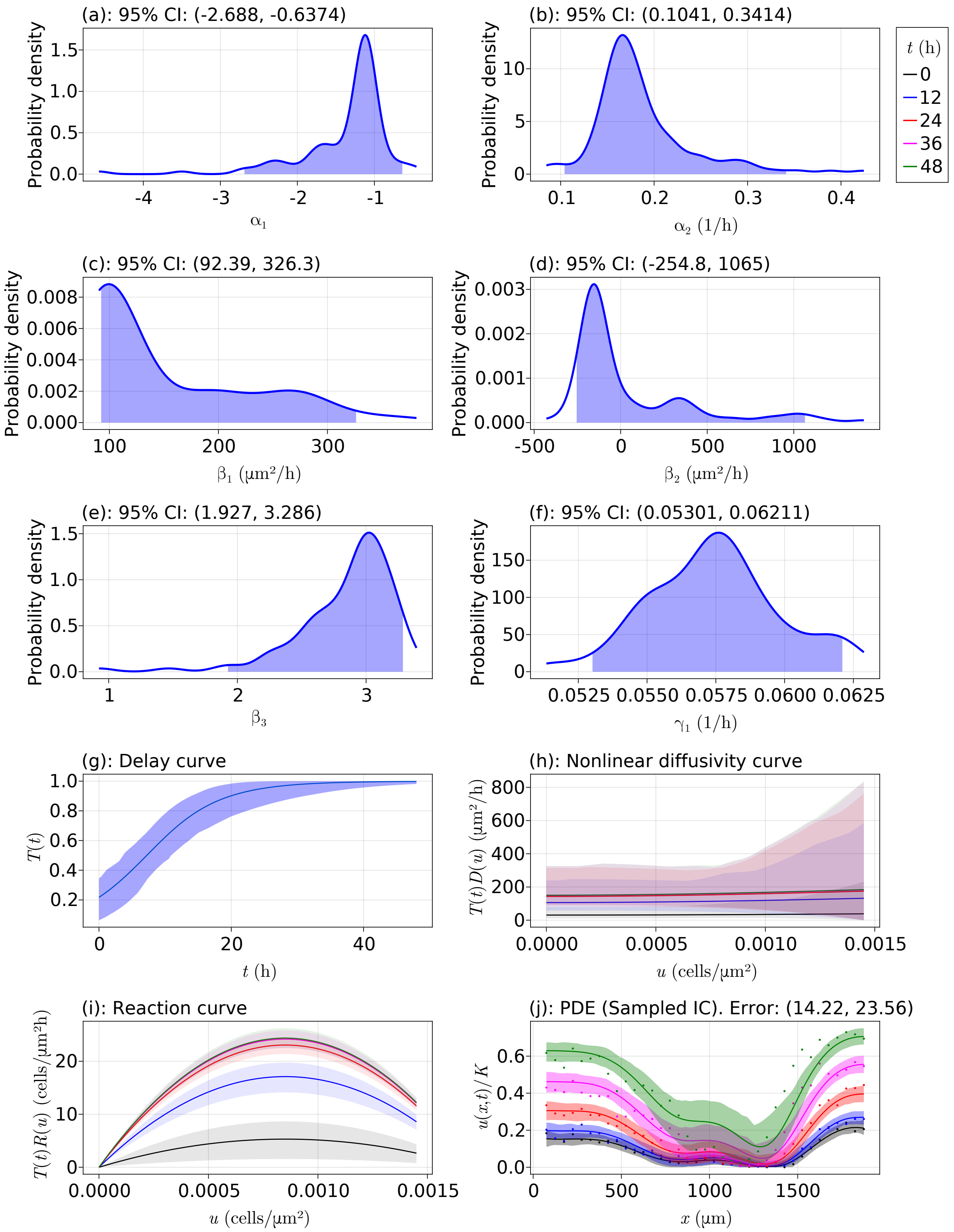

Figure P: Comparisons to Lagergren et al.'s results with 10,000 cells per well. Results for the generalised Porous-FKPP model with delay for the 10,000 cells per well data set from Table 1. In the curve plots, the different shaded regions show various values of the products of the respective mechanisms at the shown times. The shaded regions in the PDE plots represent the uncertainty at each point around the mean curve shown, and the points shown are the data from Jin et al. [1].

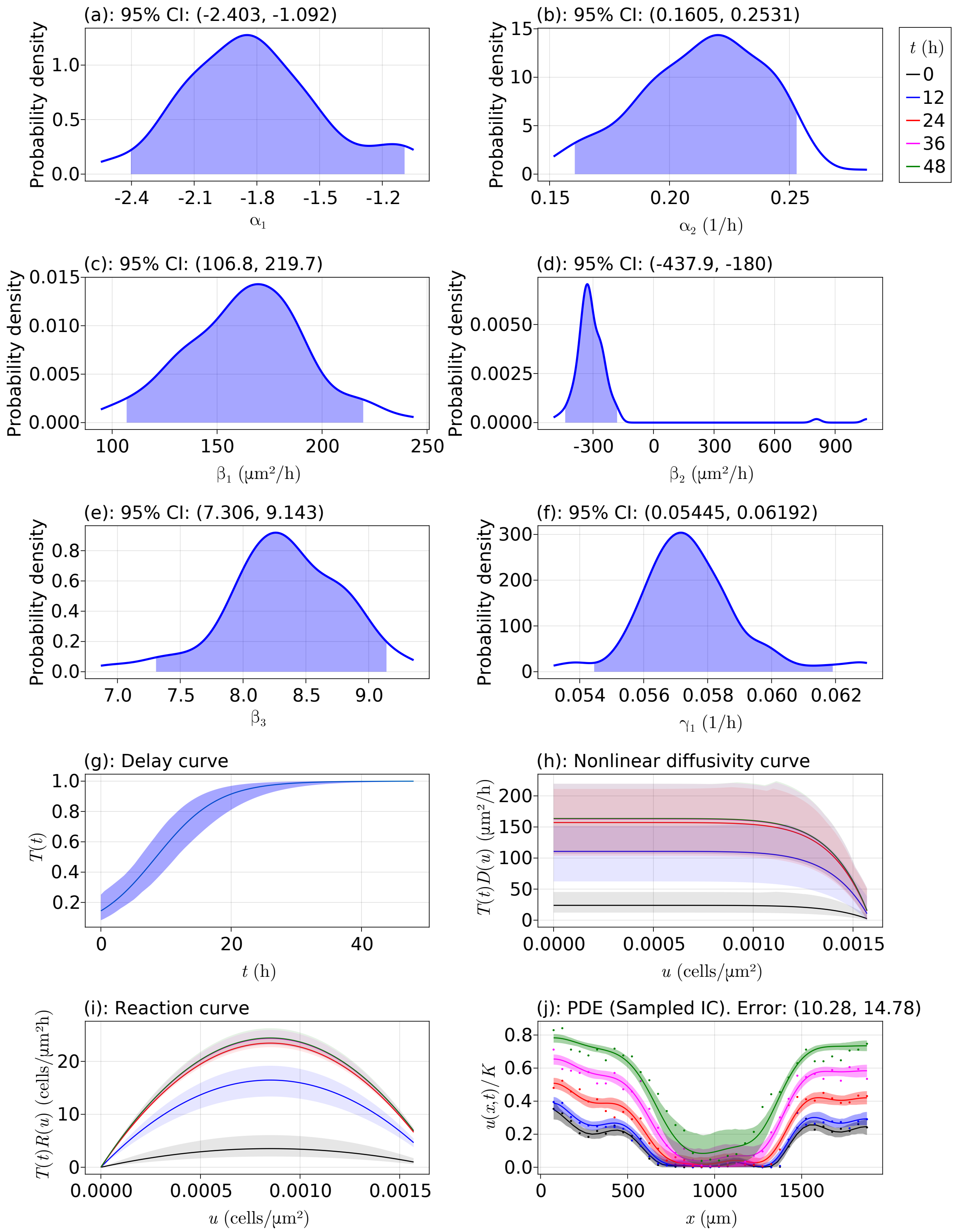

Figure Q: Comparisons to Lagergren et al.'s results with 12,000 cells per well. Results for the generalised Porous-FKPP model with delay for the 12,000 cells per well data set from Table 1. In the curve plots, the different shaded regions show various values of the products of the respective mechanisms at the shown times. The shaded regions in the PDE plots represent the uncertainty at each point around the mean curve shown, and the points shown are the data from Jin et al. [1].

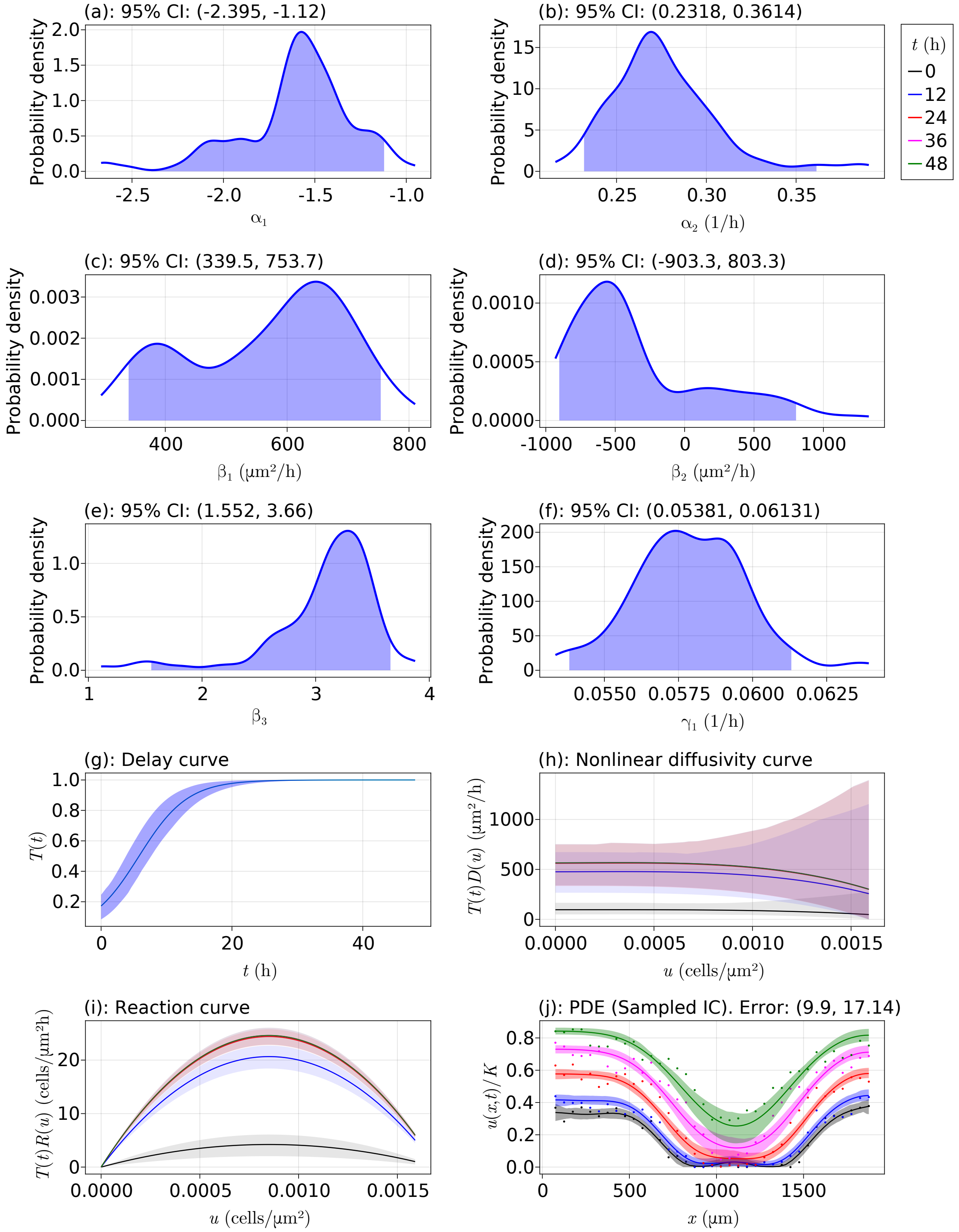

Figure R: Comparisons to Lagergren et al.'s results with 14,000 cells per well. Results for the generalised Porous-FKPP model with delay for the 14,000 cells per well data set from Table 1. In the curve plots, the different shaded regions show various values of the products of the respective mechanisms at the shown times. The shaded regions in the PDE plots represent the uncertainty at each point around the mean curve shown, and the points shown are the data from Jin et al. [1].

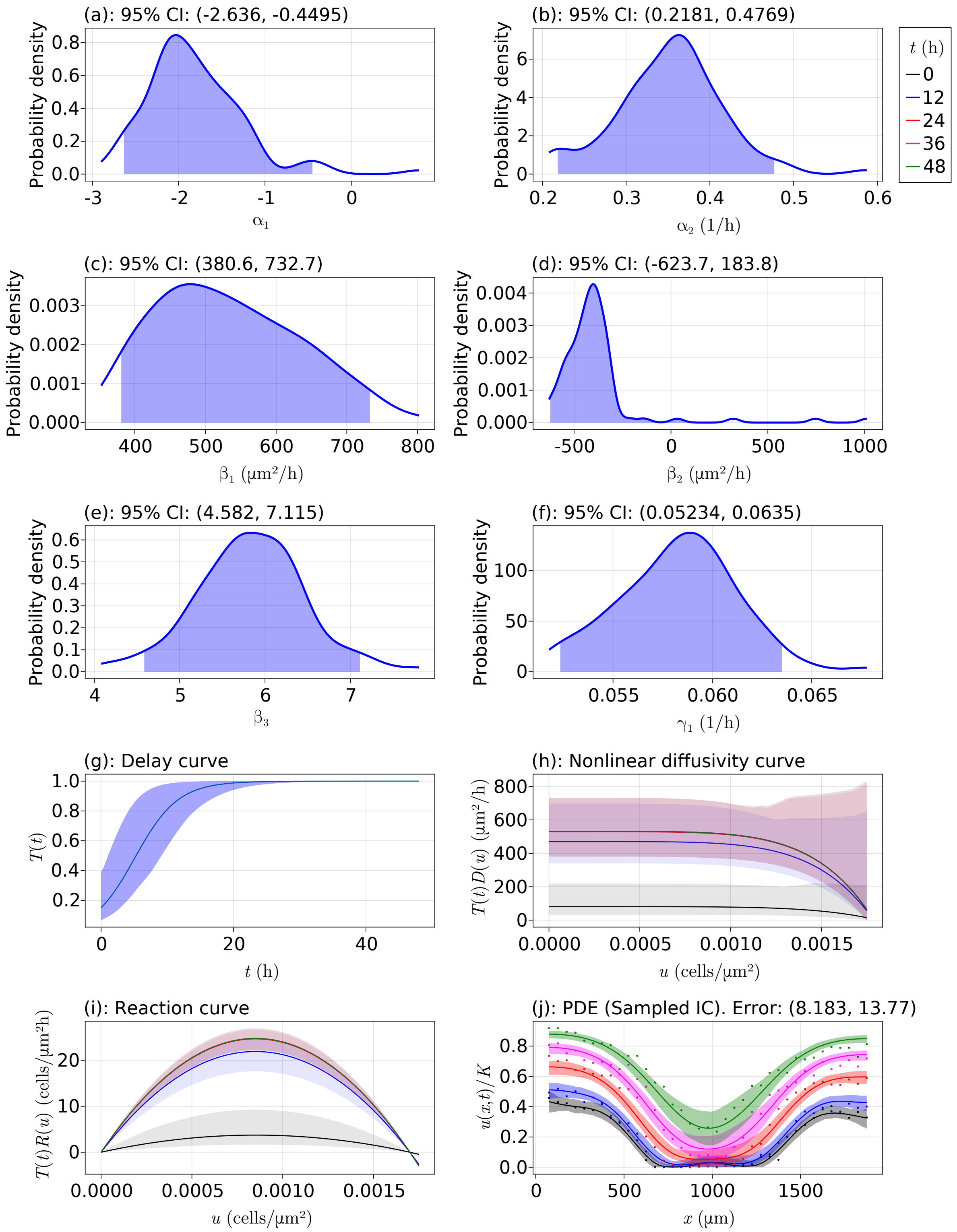

Figure S: Comparisons to Lagergren et al.'s results with with 16,000 cells per well. Results for the generalised Porous-FKPP model with delay for the 16,000 cells per well data set from Table 1 In the curve plots, the different shaded regions show various values of the products of the respective mechanisms at the shown times. The shaded regions in the PDE plots represent the uncertainty at each point around the mean curve shown, and the points shown are the data from Jin et al. [1].

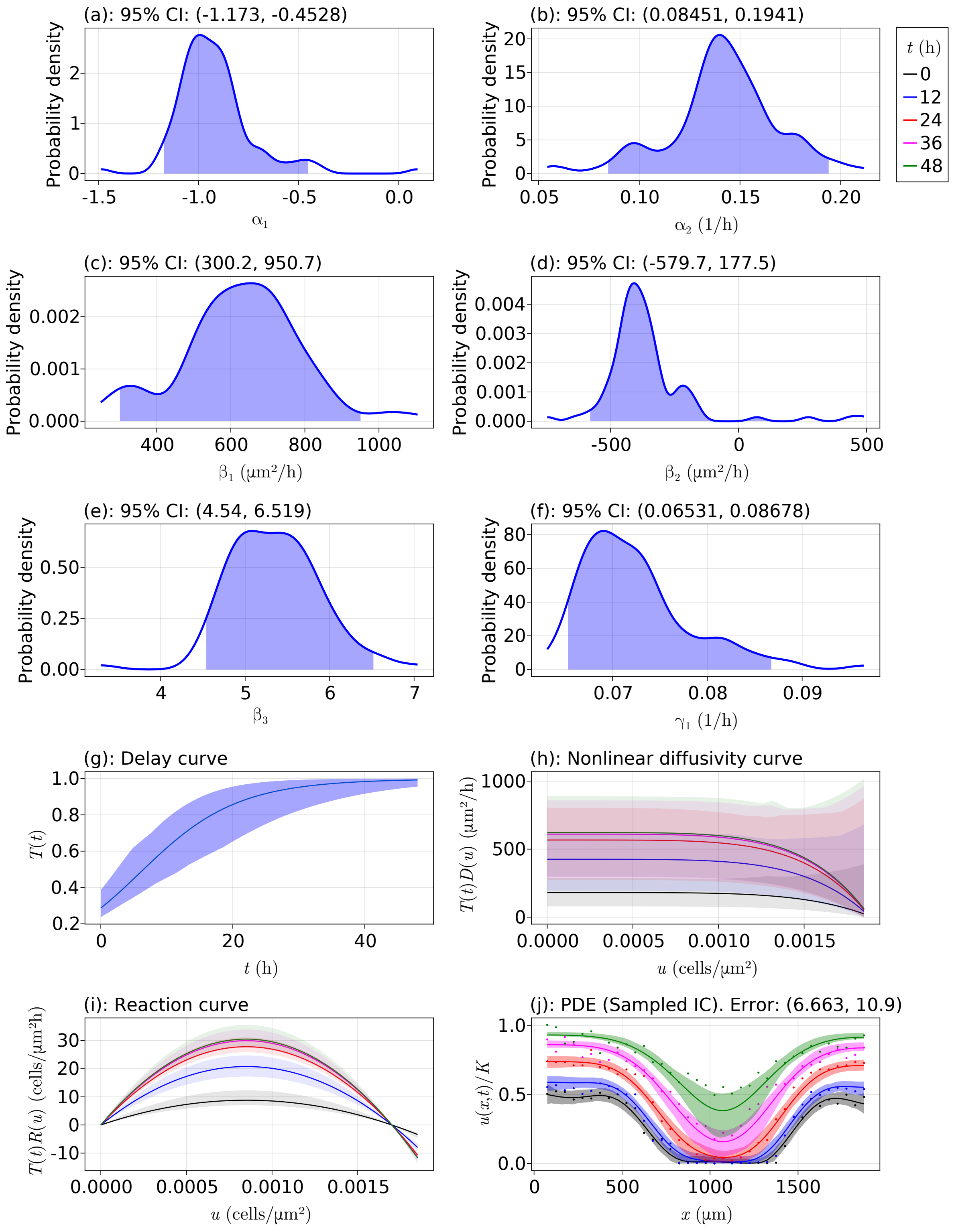

Figure T: Comparisons to Lagergren et al.'s results with 18,000 cells per well. Results for the generalised Porous-FKPP model with delay for the 18,000 cells per well data set from Table 1. In the curve plots, the different shaded regions show various values of the products of the respective mechanisms at the shown times. The shaded regions in the PDE plots represent the uncertainty at each point around the mean curve shown, and the points shown are the data from Jin et al. [1].

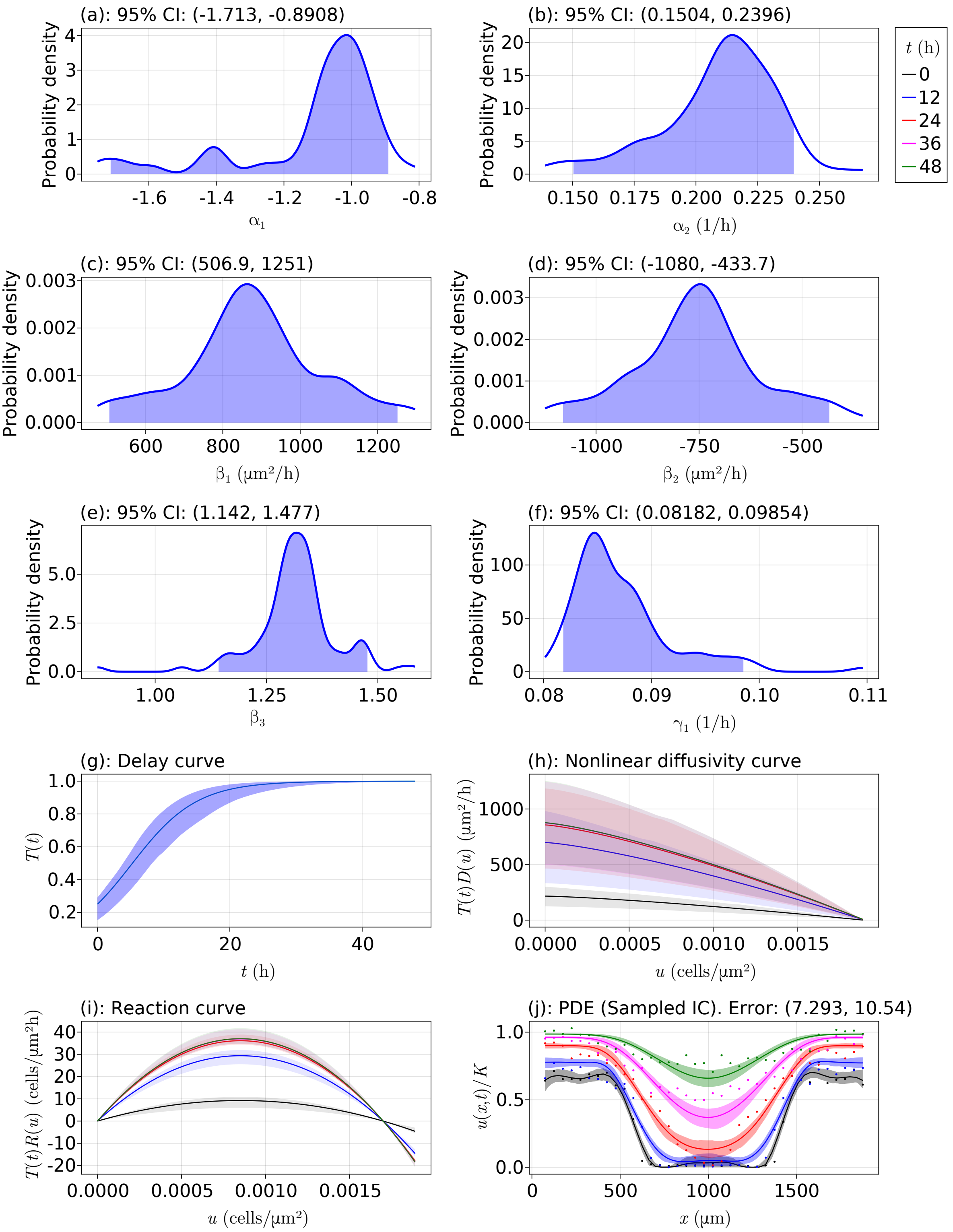

Figure U: Comparisons to Lagergren et al.'s results with 20,000 cells per well. Results for the generalised Porous-FKPP model with delay for the 20,000 cells per well data set from Table 1. In the curve plots, the different shaded regions show various values of the products of the respective mechanisms at the shown times. The shaded regions in the PDE plots represent the uncertainty at each point around the mean curve shown, and the points shown are the data from Jin et al. [1].

1. $\left(\min_{\substack{k=1,\ldots,n,\\ \ell=1,\ldots,m}} |u_{k\ell}^*|\right) \delta u \leq |u_{ij}^*| \leq \left(\max_{\substack{k=1,\ldots,n,\\ \ell=1,\ldots,m}} |u_{k\ell}^*|\right)(1-\delta u)$;
2. $\left(\min_{\substack{k=1,\ldots,n,\\ \ell=1,\ldots,m}} |\partial_t u_{k\ell}^*|\right) \delta u \leq |\partial_t u_{ij}^*| \leq \left(\max_{\substack{k=1,\ldots,n,\\ \ell=1,\ldots,m}} |\partial_t u_{k\ell}^*|\right)(1-\delta u)$;
3. $u_{ij}^* \geq 0$.

These conditions are only applied for the Gaussian process samples; none of the terms used for computing $\mathcal{L}_{\text{GLS}}$ are removed. We note that this last condition is needed since some sampled values may go below zero. With the first two conditions we can control for low density regions and for cells that are approaching the carrying capacity density, as discussed above. Those data points that do not satisfy all of these conditions are not included when computing (33).

To now explore these threshold conditions, we explore its impact on the models in (B) and (E). We use 50 bootstrap iterations for each pair $(\delta u, \delta t)$ and no optimiser restarts, and we scale the parameters such that the true parameter values are 1.0. The errors that we report from these experiments are based on the PDE solutions with the sampled initial conditions, and we only report the mean error. The results from this experiment are shown in Table C. We see that most of the minimum errors are for non-zero threshold tolerances δu, δt, with most corresponding to a 5% tolerance for the time derivatives. The global minimum in the table is 3.977, corresponding to $(\delta u, \delta t) = (0.25, 0.02)$. These results suggest that, for this model, it is more important to control for low and high density values than for the rate of evolution of these densities. This result makes sense if we consider the data in Fig A for this simulation. We see that many of the density values are near zero, and thus there are many terms in the sum defining (33) that are essentially the same. We do see that some minima are also at $\delta t = 5\%$, suggesting that we might still want to consider discarding some densities that are evolving too slowly or too fast. Overall, we see that this thresholding is worthwhile to consider in this study, but a better method would be required to more accurately estimate the optimal $(\delta u, \delta t)$ for it to be worth configuring these values given the time it takes to produce these errors.

Now let us repeat this experiment for the simulation study in Section S1.2. We now only use 10 bootstrap iterations for each pair $(\delta u, \delta t)$ and no optimiser restarts, and the parameters are scaled such that the true parameter values are 1.0. Additionally, we choose $n = m = 30$. The results for this study are shown in Table D. We see that when we introduce delay, relative to the results in Table C, thresholding is more important for the density values than for their rates of change, although there are some δu values that have a high optimal δt value, such as for the $(\delta u, \delta t) = (0.15, 0.15)$ pair. The global minimum in Table D is at $(\delta u, \delta t) = (0, 0.10)$ with an error of 3.025, showing this importance of thresholding density values, but also demonstrating that even slightly controlling for slowly/highly

δu \ δt	0%	1%	2%	3%	4%	5%	10%	15%	20%	25%
0%	**4.033**	4.118	4.158	4.086	4.116	4.123	4.147	4.277	4.205	4.116
1%	4.280	**4.078**	4.015	4.278	4.304	4.169	4.105	4.175	4.255	4.198
2%	4.206	4.265	4.234	4.211	4.305	4.045	4.196	4.272	4.366	4.310
3%	4.239	4.233	4.013	**3.998**	4.179	**4.007**	**4.103**	4.221	4.255	4.263
4%	4.180	4.183	4.152	4.130	**4.068**	4.059	4.247	**4.073**	4.355	4.412
5%	4.136	4.209	4.200	4.109	4.162	4.139	4.236	4.228	4.312	4.325
10%	4.091	4.166	4.136	4.154	4.176	4.257	4.246	4.105	4.308	**4.101**
15%	4.092	4.199	4.096	4.085	4.204	4.025	4.183	4.280	4.187	4.242
20%	4.163	4.226	4.296	4.205	4.213	4.091	4.337	4.243	4.274	4.223
25%	4.090	4.311	**3.977**	4.142	4.277	4.139	4.473	4.087	**4.057**	4.407

Table C: Results for data thresholding. Results for pairs of $(\delta u, \delta t)$. The errors reported are mean percentage errors, rounded to three decimal places, when simulating the Fisher-Kolmogorov model (B) as described in Section S1.1. The bold values show the minimum error in the column, and the underlined values show the minimum error in the row. The blue cell shows the minimum error in the whole table.

evolving densities can significantly decrease the error. We do note that the differences in these results between Table C and Table D suggest the need for future work to clarify exactly when δu and δt need to be high, and efficient procedures for estimating these tolerances would be highly beneficial for more complex data sets.

δu \ δt	0%	1%	2%	3%	4%	5%	10%	15%	20%	25%
0%	3.252	3.579	**3.334**	3.344	3.435	3.274	**3.025**	3.569	3.543	3.426
1%	3.655	3.592	3.396	**3.191**	3.285	3.106	3.432	3.813	**3.358**	3.436
2%	3.296	3.530	3.497	3.295	3.455	3.588	3.32	3.358	3.362	3.432
3%	3.510	**3.048**	3.657	3.364	3.492	**3.093**	3.344	4.024	3.629	3.531
4%	3.546	3.123	3.874	3.726	3.297	3.212	3.367	3.387	3.670	3.408
5%	**3.160**	3.446	3.475	3.467	3.626	3.810	3.361	3.609	3.651	**3.262**
10%	3.525	3.294	3.502	3.607	**3.278**	3.365	3.641	3.572	3.446	3.491
15%	3.312	3.745	3.373	3.328	3.580	3.664	3.602	**3.258**	3.573	3.519
20%	3.820	3.396	3.348	3.437	3.647	3.255	3.206	3.430	3.583	3.469
25%	3.494	3.298	3.408	3.536	3.747	3.239	3.629	3.594	3.454	3.341

Table D: Results for data thresholding. Results for pairs of $(\delta u, \delta t)$. The errors reported are mean percentage errors when simulating the Fisher-Kolmogorov model with delay as described in Section S1.2. The bold values show the minimum error in the column, and the underlined values show the minimum error in the row. The blue cell shows the minimum error in the whole table.

S5 PDE truncation error

In this section we analyse how different choices of spatial discretisation, and the associated truncation error, affects the inferences made from the bootstrapping procedure. We perform this analysis by considering the results when using $N_p = 500$, 250, 125, or 50 equally-spaced spatial gridpoints; the temporal discretisation is chosen adaptively and hence no analysis of it will be presented here. We list below four tables, Table E–H, that compare results with $N_p = 500, 250, 125,$ and 50, respectively. Note that Table

E is the same as Table 1 to facilitate the comparison. We see from these tables that the results are essentially the same across each grid size, with the same model selected each time. For this best model, model 1, we show the confidence intervals for all the coefficients and for the PDE errors for each N_p and each data set in Fig V. This figure shows that there are virtually no differences in the results. Most remarkably, we have the following runtimes for producing these results:

- $N_p = 500$: 31 hours and 40 minutes.
- $N_p = 250$: 4 hours and 37 minutes.
- $N_p = 125$: 1 hour and 33 minutes.
- $N_p = 50$: 56 minutes and 7 seconds.

These simulation times are produced on a Windows 10 64-bit machine with an i7-8700K CPU @ 3.70 GHz, 32 GB of RAM, and a GTX 1070 Ti, and no parallel computing is used for bootstrapping. We could therefore produce the required results in just under an hour, for all data sets and for all models, without exploiting parallelism. Hence, we see that the net error introduced by solving these PDEs across a wide range of parameter sets is relatively insensitive to the truncation error introduced through spatial discretisation. In practice, then, we could find the results with $N_p = 50$ and $N_p = 125$ and, if there is little improvement in the $N_p = 125$ case, we could be satisfied with the $N_p = 50$ results rather than be concerned about the impact of the spatial truncation.

	Models				
	Model 1	Model 2	Model 3	Model 4	Model 5
**10,000 cells per well**					
PDE Error (%)	(20.864, 32.123)	(13.943, 23.048)	(21.158, 32.887)	(14.554, 23.934)	(14.219, 23.564)
α_1	—	(−3.456, −0.217)	—	(−3.863, −0.372)	(−2.688, −0.637)
α_2 (h^{-1})	—	(0.091, 0.373)	—	(0.084, 0.402)	(0.104, 0.341)
β_1 (µm^2 h^{-1})	(54.396, 289.822)	(51.93, 365.923)	—	—	(92.394, 326.322)
β_2 (µm^2 h^{-1})	—	—	(20.608, 979.03)	(13.828, 849.614)	(−254.821, 1065.03)
β_3	—	—	—	—	(1.927, 3.286)
γ_1 (h^{-1})	(0.045, 0.052)	(0.054, 0.063)	(0.045, 0.052)	(0.053, 0.065)	(0.053, 0.062)
$\mathbb{P}(E_1)$	0.00	0.96	0.0	0.07	0.04
$\mathbb{P}(E_2)$	0.01	0.00	0.0	0.03	0.23
$\mathbb{P}(E_3)$	0.99	0.04	1.0	0.90	0.73
**12,000 cells per well**					
PDE Error (%)	(15.736, 21.384)	(10.104, 14.547)	(15.23, 21.331)	(9.747, 14.411)	(10.282, 14.783)
α_1	—	(−3.088, −0.854)	—	(−3.055, −0.826)	(−2.403, −1.092)
α_2 (h^{-1})	—	(0.133, 0.307)	—	(0.13, 0.303)	(0.16, 0.253)
β_1 (µm^2 h^{-1})	(91.735, 193.201)	(117.181, 222.334)	—	—	(106.845, 219.685)
β_2 (µm^2 h^{-1})	—	—	(207.898, 584.976)	(194.568, 531.537)	(−437.945, −179.991)
β_3	—	—	—	—	(7.306, 9.143)
γ_1 (h^{-1})	(0.044, 0.049)	(0.055, 0.062)	(0.044, 0.048)	(0.054, 0.061)	(0.054, 0.062)
$\mathbb{P}(E_1)$	0.00	1.00	0.00	0.00	0.16
$\mathbb{P}(E_2)$	0.00	0.00	0.00	0.00	0.49
$\mathbb{P}(E_3)$	1.00	0.00	1.00	1.00	0.35
**14,000 cells per well**					
PDE Error (%)	(11.794, 14.811)	(10.147, 14.635)	(10.598, 16.571)	(9.996, 15.573)	(9.9, 17.135)
α_1	—	(−2.858, −0.866)	—	(−2.588, −0.744)	(−2.395, −1.12)
α_2 (h^{-1})	—	(0.199, 0.401)	—	(0.19, 0.401)	(0.232, 0.361)
β_1 (µm^2 h^{-1})	(423.353, 689.093)	(452.419, 763.335)	—	—	(339.505, 753.689)
β_2 (µm^2 h^{-1})	—	—	(956.516, 2020.74)	(841.97, 2012.11)	(−903.293, 803.306)
β_3	—	—	—	—	(1.552, 3.66)
γ_1 (h^{-1})	(0.047, 0.052)	(0.054, 0.061)	(0.047, 0.052)	(0.052, 0.059)	(0.054, 0.061)
$\mathbb{P}(E_1)$	0.05	0.84	0.04	0.01	0.20
$\mathbb{P}(E_2)$	0.04	0.09	0.01	0.01	0.20
$\mathbb{P}(E_3)$	0.91	0.07	0.95	0.98	0.60
**16,000 cells per well**					
PDE Error (%)	(10.648, 14.259)	(8.407, 12.702)	(10.217, 15.381)	(8.876, 14.332)	(8.183, 13.768)
α_1	—	(−2.256, 0.022)	—	(−2.17, −0.1)	(−2.636, −0.449)
α_2 (h^{-1})	—	(0.097, 0.335)	—	(0.102, 0.34)	(0.218, 0.477)
β_1 (µm^2 h^{-1})	(382.49, 647.908)	(434.293, 727.624)	—	—	(380.617, 732.718)
β_2 (µm^2 h^{-1})	—	—	(808.965, 1581.16)	(728.143, 1549.18)	(−623.709, 183.84)
β_3	—	—	—	—	(4.582, 7.115)
γ_1 (h^{-1})	(0.049, 0.055)	(0.056, 0.067)	(0.048, 0.054)	(0.055, 0.065)	(0.052, 0.064)
$\mathbb{P}(E_1)$	0.01	0.92	0.00	0.07	0.15
$\mathbb{P}(E_2)$	0.00	0.01	0.00	0.00	0.22
$\mathbb{P}(E_3)$	0.99	0.07	1.00	0.93	0.63
**18,000 cells per well**					
PDE Error (%)	(7.949, 10.907)	(6.609, 8.923)	(8.772, 12.14)	(7.152, 9.74)	(6.663, 10.902)
α_1	—	(−1.45, 0.066)	—	(−1.436, −0.022)	(−1.173, −0.453)
α_2 (h^{-1})	—	(0.059, 0.236)	—	(0.056, 0.207)	(0.085, 0.194)
β_1 (µm^2 h^{-1})	(433.393, 760.833)	(470.944, 922.542)	—	—	(300.168, 950.728)
β_2 (µm^2 h^{-1})	—	—	(655.963, 1614.74)	(561.39, 1724.61)	(−579.707, 177.486)
β_3	—	—	—	—	(4.54, 6.519)
γ_1 (h^{-1})	(0.054, 0.061)	(0.064, 0.088)	(0.053, 0.06)	(0.064, 0.09)	(0.065, 0.087)
$\mathbb{P}(E_1)$	0.04	0.76	0.00	0.00	0.32
$\mathbb{P}(E_2)$	0.01	0.11	0.00	0.00	0.07
$\mathbb{P}(E_3)$	0.95	0.13	1.00	1.00	0.61
**20,000 cells per well**					
PDE Error (%)	(8.76, 11.287)	(7.206, 9.61)	(10.896, 14.895)	(8.211, 11.926)	(7.293, 10.536)
α_1	—	(−4.544, −1.961)	—	(−2.402, −1.721)	(−1.713, −0.891)
α_2 (h^{-1})	—	(0.301, 0.555)	—	(0.269, 0.384)	(0.15, 0.24)
β_1 (µm^2 h^{-1})	(401.865, 664.394)	(443.875, 852.295)	—	—	(506.936, 1251.44)
β_2 (µm^2 h^{-1})	—	—	(628.934, 1170.63)	(627.656, 1461.9)	(−1080.17, −433.677)
β_3	—	—	—	—	(1.142, 1.477)
γ_1 (h^{-1})	(0.069, 0.077)	(0.083, 0.093)	(0.072, 0.084)	(0.086, 0.099)	(0.082, 0.099)
$\mathbb{P}(E_1)$	0.00	0.84	0.00	0.00	0.26
$\mathbb{P}(E_2)$	0.00	0.09	0.00	0.00	0.09
$\mathbb{P}(E_3)$	1.00	0.07	1.00	1.00	0.65

Table E: Model selection results for the five models considered with $N_p = 500$. Interval estimates, PDE errors, and model selection results for the models in (2)–(6) when applied to each data set from Jin et al. [1]. See the paper for more details. The time taken to produce these results is 31 hours and 40 minutes.

	Model 1	Model 2	Model 3	Model 4	Model 5
**10,000 cells per well**					
PDE Error (%)	$(21.2, 32.203)$	$(14.442, 23.24)$	$(21.012, 32.757)$	$(14.767, 24.179)$	$(14.361, 23.758)$
α_1	—	$(-3.422, -0.212)$	—	$(-3.919, -0.383)$	$(-2.585, -0.561)$
α_2 (h^{-1})	—	$(0.092, 0.373)$	—	$(0.082, 0.406)$	$(0.102, 0.344)$
β_1 (µm^2 h^{-1})	$(52.953, 288.804)$	$(49.109, 363.554)$	—	—	$(92.393, 304.617)$
β_2 (µm^2 h^{-1})	—	—	$(16.398, 965.198)$	$(12.471, 841.631)$	$(-288.928, 1031.84)$
β_3	—	—	—	—	$(1.847, 3.313)$
γ_1 (h^{-1})	$(0.045, 0.052)$	$(0.054, 0.063)$	$(0.045, 0.052)$	$(0.053, 0.064)$	$(0.053, 0.062)$
$\mathbb{P}(E_1)$	0.00	0.96	0.00	0.07	0.04
$\mathbb{P}(E_2)$	0.01	0.00	0.00	0.05	0.24
$\mathbb{P}(E_3)$	0.99	0.04	1.00	0.88	0.72
**12,000 cells per well**					
PDE Error (%)	$(15.624, 21.434)$	$(9.883, 14.506)$	$(15.193, 21.757)$	$(9.698, 14.355)$	$(10.119, 14.481)$
α_1	—	$(-30.095, -0.857)$	—	$(-3.064, -0.852)$	$(-2.403, -1.096)$
α_2 (h^{-1})	—	$(0.132, 0.307)$	—	$(0.129, 0.303)$	$(0.16, 0.253)$
β_1 (µm^2 h^{-1})	$(93.032, 194.788)$	$(117.614, 223.516)$	—	—	$(108.136, 223.539)$
β_2 (µm^2 h^{-1})	—	—	$(210.106, 590.644)$	$(188.792, 543.41)$	$(-437.724, 92.923)$
β_3	—	—	—	—	$(7.368, 9.038)$
γ_1 (h^{-1})	$(0.044, 0.049)$	$(0.055, 0.062)$	$(0.044, 0.048)$	$(0.054, 0.06)$	$(0.055, 0.062)$
$\mathbb{P}(E_1)$	(0.00	1.00	0.00	0.00	0.19
$\mathbb{P}(E_2)$	0.00	0.00	0.00	0.00	0.43
$\mathbb{P}(E_3)$	1.00	0.00	1.00	1.00	0.38
**14,000 cells per well**					
PDE Error (%)	$(11.891, 14.843)$	$(10.144, 14.593)$	$(10.689, 17.045)$	$(10.294, 15.871)$	$(9.881, 17.121)$
α_1	—	$(-2.849, -0.861)$	—	$(-2.585, -0.724)$	$(-2.112, -1.124)$
α_2 (h^{-1})	—	$(0.199, 0.4)$	—	$(0.194, 0.401)$	$(0.236, 0.326)$
β_1 (µm^2 h^{-1})	$(422.34, 689.432)$	$(451.335, 763.513)$	—	—	$(339.428, 762.324)$
β_2 (µm^2 h^{-1})	—	—	$(954.838, 2028.26)$	$(840.05, 2011.48)$	$(-902.36, 859.7)$
β_3	—	—	—	—	$(2.064, 3.607)$
γ_1 (h^{-1})	$(0.047, 0.052)$	$(0.054, 0.061)$	$(0.047, 0.052)$	$(0.052, 0.06)$	$(0.054, 0.061)$
$\mathbb{P}(E_1)$	0.04	0.88	0.04	0.01	0.22
$\mathbb{P}(E_2)$	0.05	0.06	0.00	0.01	0.18
$\mathbb{P}(E_3)$	0.91	0.06	0.96	0.98	0.60
**16,000 cells per well**					
PDE Error (%)	$(10.381, 14.117)$	$(81.52, 12.437)$	$(10.212, 15.569)$	$(8.827, 13.935)$	$(8.166, 13.329)$
α_1	—	$(-2.261, -0.059)$	—	$(-2.168, -0.097)$	$(-2.649, -0.449)$
α_2 (h^{-1})	—	$(0.098, 0.335)$	—	$(0.103, 0.334)$	$(0.218, 0.477)$
β_1 (µm^2 h^{-1})	$(383.776, 648.676)$	$(434.816, 728.194)$	—	—	$(381.006, 730.965)$
β_2 (µm^2 h^{-1})	—	—	$(814.816, 1559.22)$	$(733.076, 1525.23)$	$(-623.22, 185.818)$
β_3	—	—	—	—	$(4.584, 7.116)$
γ_1 (h^{-1})	$(0.049, 0.055)$	$(0.056, 0.067)$	$(0.048, 0.054)$	$(0.055, 0.065)$	$(0.052, 0.064)$
$\mathbb{P}(E_1)$	0.00	0.91	0.00	0.07	0.18
$\mathbb{P}(E_2)$	0.01	0.02	0.00	0.00	0.18
$\mathbb{P}(E_3)$	0.99	0.07	1.00	0.93	0.64
**18,000 cells per well**					
PDE Error (%)	$(7.843, 10.923)$	$(6.618, 8.974)$	$(8.681, 12.34)$	$(7.03, 9.73)$	$(6.613, 10.348)$
α_1	—	$(-1.45, 0.073)$	—	$(-1.432, -0.036)$	$(-1.169, -0.452)$
α_2 (h^{-1})	—	$(0.06, 0.236)$	—	$(0.054, 0.208)$	$(0.085, 0.193)$
β_1 (µm^2 h^{-1})	$(434.088, 761.277)$	$(471.109, 922.808)$	—	—	$(300.572, 950.847)$
β_2 (µm^2 h^{-1})	—	—	$(658.218, 1616.97)$	$(557.901, 1706.56)$	$(-579.641, 170.527)$
β_3	—	—	—	—	$(4.539, 6.616)$
γ_1 (h^{-1})	$(0.054, 0.061)$	$(0.064, 0.088)$	$(0.053, 0.06)$	$(0.063, 0.09)$	$90.065, 0.087)$
$\mathbb{P}(E_1)$	0.05	0.77	0.00	0.00	0.32
$\mathbb{P}(E_2)$	0.01	0.10	0.00	0.00	0.09
$\mathbb{P}(E_3)$	0.94	0.13	1.00	1.00	0.59
**20,000 cells per well**					
PDE Error (%)	$(8.897, 11.377)$	$(7.092, 9.527)$	$(11.039, 14.935)$	$(8.506, 12.046)$	$(7.28, 10.433)$
α_1	—	$(-4.544, -1.965)$	—	$(-2.402, -1.717)$	$(-1.712, -0.89)$
α_2 (h^{-1})	—	$(0.301, 0.539)$	—	$(0.262, 0.381)$	$(0.151, 0.238)$
β_1 (µm^2 h^{-1})	$(404.252, 667.269)$	$(446.18, 855.255)$	—	—	$(509.336, 1287.92)$
β_2 (µm^2 h^{-1})	—	—	$(630.293, 1173.08)$	$(635.53, 1468.25)$	$(-1112.18, -435.678)$
β_3	—	—	—	—	$(1.151, 1.477)$
γ_1 (h^{-1})	$(0.069, 0.077)$	$(0.083, 0.093)$	$(0.072, 0.084)$	$(0.086, 0.101)$	$(0.082, 0.098)$
$\mathbb{P}(E_1)$	0.00	0.83	0.00	0.00	0.28
$\mathbb{P}(E_2)$	0.00	0.08	0.00	0.00	0.11
$\mathbb{P}(E_3)$	1.00	0.09	1.00	1.00	0.61

Table F: Model selection results for the five models considered with $N_p = 250$. Interval estimates, PDE errors, and model selection results for the models in (2)–(6) when applied to each data set from Jin et al. [1]. See the paper for more details. The time taken to produce these results is 4 hours and 37 minutes.

	Models				
	Model 1	Model 2	Model 3	Model 4	Model 5
**10,000 cells per well**					
PDE Error (%)	$(21.123, 32.054)$	$(14.392, 23.055)$	$(21.262, 32.608)$	$(14.75, 24.103)$	$(14.614, 23.271)$
α_1	—	$(-3.753, -0.195)$	—	$(-3.839, -0.378)$	$(-2.615, -0.559)$
α_2 (h^{-1})	—	$(0.089, 0.379)$	—	$(0.083, 0.403)$	$(0.107, 0.324)$
β_1 ($\mu\mathrm{m}^2\,\mathrm{h}^{-1}$)	$(55.349, 290.935)$	$(49.757, 365.77)$	—	—	$(92.387, 311.556)$
β_2 ($\mu\mathrm{m}^2\,\mathrm{h}^{-1}$)	—	—	$(26.938, 976.404)$	$(16.577, 951.623)$	$(-283.614, 862.542)$
β_3	—	—	—	—	$(1.825, 3.312)$
γ_1 (h^{-1})	$(0.045, 0.052)$	$(0.054, 0.064)$	$(0.045, 0.052)$	$(0.054, 0.064)$	$(0.053, 0.062)$
$\mathbb{P}(E_1)$	0.00	0.96	0.00	0.07	0.06
$\mathbb{P}(E_2)$	0.01	0.00	0.00	0.04	0.25
$\mathbb{P}(E_3)$	0.99	0.04	1.00	0.89	0.69
**12,000 cells per well**					
PDE Error (%)	$(15.609, 20.958)$	$(9.769, 14.127)$	$(15.318, 21.31)$	$(.491, 14.802)$	$(.899, 14.702)$
α_1	—	$(-3.098, -0.854)$	—	$(-3.071, -0.88)$	$(-2.414, -1.115)$
α_2 (h^{-1})	—	$(0.132, 0.308)$	—	$(0.136, 0.304)$	$(0.159, 0.256)$
β_1 ($\mu\mathrm{m}^2\,\mathrm{h}^{-1}$)	$(91.584, 193.238)$	$(116.435, 222.262)$	—	—	$(107.951, 220.655)$
β_2 ($\mu\mathrm{m}^2\,\mathrm{h}^{-1}$)	—	—	$(208.632, 583.982)$	$(193.116, 554.899)$	$(-443.453, -90.742)$
β_3	—	—	—	—	$(7.221, 9.236)$
γ_1 (h^{-1})	$(0.044, 0.049)$	$(0.055, 0.062)$	$(0.044, 0.048)$	$(0.054, 0.061)$	$(0.055, 0.062)$
$\mathbb{P}(E_1)$	0.00	0.98	0.00	0.00	0.108
$\mathbb{P}(E_2)$	0.00	0.02	0.00	0.00	0.50
$\mathbb{P}(E_3)$	1.00	0.00	1.00	1.00	0.32
**14,000 cells per well**					
PDE Error (%)	$(11.806, 15.038)$	$(10.201, 14.636)$	$(10.7, 16.654)$	$(10.319, 15.86)$	$(9.915, 16.866)$
α_1	—	$(-2.853, -0.865)$	—	$(-2.577, -0.74)$	$(-2.181, -1.128)$
α_2 (h^{-1})	—	$(0.199, 0.4)$	—	$(0.195, 0.398)$	$(0.232, 0.338)$
β_1 ($\mu\mathrm{m}^2\,\mathrm{h}^{-1}$)	$(423.818, 689.947)$	$(452.78, 763.137)$	—	—	$(342.857, 760.395)$
β_2 ($\mu\mathrm{m}^2\,\mathrm{h}^{-1}$)	—	—	$(954.855, 2030.49)$	$(842.721, 2010.96)$	$(-897.266, 837.937)$
β_3	—	—	—	—	$(2.06, 3.56)$
γ_1 (h^{-1})	$(0.047, 0.052)$	$(0.054, 0.061)$	$(0.047, 0.052)$	$(0.052, 0.059)$	$(0.054, 0.061)$
$\mathbb{P}(E_1)$	0.05	0.88	0.04	0.01	0.22
$\mathbb{P}(E_2)$	0.04	0.07	0.01	0.01	0.19
$\mathbb{P}(E_3)$	0.91	0.05	0.95	0.98	0.59
**16,000 cells per well**					
PDE Error (%)	$(10.623, 14.231)$	$(8.287, 12.4)$	$(10.179, 15.424)$	$(8.564, 13.759)$	$(8.522, 13.732)$
α_1	—	$(-2.26, 0.022)$	—	$(-2.169, -0.104)$	$(-2.638, -0.444)$
α_2 (h^{-1})	—	$(0.097, 0.336)$	—	$(0.102, 0.347)$	$(0.211, 0.477)$
β_1 ($\mu\mathrm{m}^2\,\mathrm{h}^{-1}$)	$(382.172, 647.945)$	$(433.674, 727.565)$	—	—	$(380.731, 735.064)$
β_2 ($\mu\mathrm{m}^2\,\mathrm{h}^{-1}$)	—	—	$(791.717, 1557.42)$	$(728.252, 1531.33)$	$(-624.559, 188.586)$
β_3	—	—	—	—	$(4.577, 7.114)$
γ_1 (h^{-1})	$(0.049, 0.055)$	$(0.056, 0.067)$	$(0.048, 0.054)$	$(0.055, 0.065)$	$(0.052, 0.064)$
$\mathbb{P}(E_1)$	0.01	0.91	0.00	0.87	0.17
$\mathbb{P}(E_2)$	0.00	0.01	0.00	0.00	0.19
$\mathbb{P}(E_3)$	0.99	0.08	1.00	0.93	0.64
**18,000 cells per well**					
PDE Error (%)	$(7.924, 10.89)$	$(6.666, 8.521)$	$(8.391, 12.335)$	$(6.961, 9.697)$	$(6.566, 10.277)$
α_1	—	$(-1.45, 0.07)$	—	$(-1.435, -0.03)$	$(-1.169, -0.452)$
α_2 (h^{-1})	—	$(0.061, 0.236)$	—	$(0.053, 0.196)$	$(0.085, 0.19)$
β_1 ($\mu\mathrm{m}^2\,\mathrm{h}^{-1}$)	$(433.372, 761.251)$	$(470.508, 922.76)$	—	—	$(299.552, 950.87)$
β_2 ($\mu\mathrm{m}^2\,\mathrm{h}^{-1}$)	—	—	$(652.427, 1615.53)$	$(535.967, 1715.56)$	$(-602.8, 173.175)$
β_3	—	—	—	—	$(4.54, 6.521)$
γ_1 (h^{-1})	$(0.054, 0.061)$	$(0.064, 0.087)$	$(0.053, 0.06)$	$(0.064, 0.092)$	$(0.065, 0.087)$
$\mathbb{P}(E_1)$	0.05	0.76	0.00	0.00	0.33
$\mathbb{P}(E_2)$	0.01	0.11	0.00	0.00	0.07
$\mathbb{P}(E_3)$	0.94	0.13	1.00	1.00	0.60
**20,000 cells per well**					
PDE Error (%)	$(8.881, 11.208)$	$(7.159, 9.53)$	$(11.379, 15.086)$	$(8.486, 12.023)$	$(7.52, 10.372)$
α_1	—	$(-4.559, -1.964)$	—	$(-2.5, -1.768)$	$(-1.72, -0.896)$
α_2 (h^{-1})	—	$(0.301, 0.552)$	—	$(0.265, 0.391)$	$(0.149, 0.24)$
β_1 ($\mu\mathrm{m}^2\,\mathrm{h}^{-1}$)	$(401.357, 663.987)$	$(442.131, 851.148)$	—	—	$(504.775, 1255.85)$
β_2 ($\mu\mathrm{m}^2\,\mathrm{h}^{-1}$)	—	—	$(631.029, 1172.92)$	$(626.089, 1452.64)$	$(-1088.07, -430.534)$
β_3	—	—	—	—	$(1.156, 1.483)$
γ_1 (h^{-1})	$(0.069, 0.077)$	$(0.083, 0.93)$	$(0.072, 0.084)$	$(0.086, 0.101)$	$(0.082, 0.098)$
$\mathbb{P}(E_1)$	(0.00	0.80	0.00	0.00	0.27
$\mathbb{P}(E_2)$	0.00	0.10	0.00	0.00	0.09
$\mathbb{P}(E_3)$	1.00	0.10	1.00	1.00	0.64

Table G: Model selection results for the five models considered with $N_p = 125$. Interval estimates, PDE errors, and model selection results for the models in (2)–(6) when applied to each data set from Jin et al. [1]. See the paper for more details. The time taken to produce these results is 1 hour and 33 minutes.

	Models				
	Model 1	Model 2	Model 3	Model 4	Model 5
			10,000 cells per well		
PDE Error (%)	$(20.841, 32.359)$	$(14.099, 22.278)$	$(21.9, 32.748)$	$(14.408, 24.14)$	$(13.81, 24.611)$
α_1	—	$(-3.826, -0.248)$	—	$(-3.953, -0.261)$	$(-2.85, -0.639)$
α_2 (h^{-1})	—	$(0.085, 0.39)$	—	$(0.082, 0.406)$	$(0.106, 0.358)$
β_1 (µm^2 h^{-1})	$(60.011, 296.129)$	$(71.496, 368.451)$	—	—	$(92.504, 325.704)$
β_2 (µm^2 h^{-1})	—	—	$(42.183, 965.966)$	$(19.601, 862.975)$	$(-300.098, 1081.19)$
β_3	—	—	—	—	$(1.76, 3.316)$
γ_1 (h^{-1})	$(0.045, 0.052)$	$(0.054, 0.064)$	$(0.045, 0.052)$	$(0.054, 0.065)$	$(0.053, 0.062)$
$\mathbb{P}(E_1)$	0.00	0.96	0.00	0.06	0.04
$\mathbb{P}(E_2)$	0.00	0.00	0.00	0.04	0.24
$\mathbb{P}(E_3)$	1.00	0.04	1.00	0.90	0.72
			12,000 cells per well		
PDE Error (%)	$(15.566, 21.498)$	$(9.952, 14.49)$	$(15.914, 22.565)$	$(10.463, 15.341)$	$(9.853, 14.737)$
α_1	—	$(-3.072, 0.891)$	—	$(-2.991, -0.852)$	$(-2.397, -1.104)$
α_2 (h^{-1})	—	$(0.135, 0.306)$	—	$(0.134, 0.299)$	$(0.16, 0.255)$
β_1 (µm^2 h^{-1})	$(95.843, 194.843)$	$(119.06, 226.051)$	—	—	$(108.728, 222.842)$
β_2 (µm^2 h^{-1})	—	—	$(253.376, 592.556)$	$(213.861, 547.584)$	$(-448.875, -147.375)$
β_3	—	—	—	—	$(7.37, 9.097)$
γ_1 (h^{-1})	$(0.044, 0.049)$	$(0.055, 0.062)$	$(0.044, 0.048)$	$(0.054, 0.061)$	$(0.054, 0.062)$
$\mathbb{P}(E_1)$	0.00	0.99	0.00	0.00	0.17
$\mathbb{P}(E_2)$	0.00	0.01	0.00	0.00	0.47
$\mathbb{P}(E_3)$	1.00	0.00	1.00	1.00	0.36
			14,000 cells per well		
PDE Error (%)	$(12.043, 14.8)$	$(10.111, 14.746)$	$(10.932, 15.963)$	$(10.517, 15.917)$	$(10.205, 16.984)$
α_1	—	$(-2.852, -0.867)$	—	$(-2.579, -0.717)$	$(-2.098, -1.122)$
α_2 (h^{-1})	—	$(0.199, 0.401)$	—	$(0.194, 0.412)$	$(0.236, 0.331)$
β_1 (µm^2 h^{-1})	$(424.702, 689.249)$	$(453.057, 763.85)$	—	—	$(342.357, 761.589)$
β_2 (µm^2 h^{-1})	—	—	$(973.031, 2022.92)$	$(852.553, 2002.69)$	$(-896.467, 915.056)$
β_3	—	—	—	—	$(1.75, 3.663)$
γ_1 (h^{-1})	$(0.047, 0.052)$	$(0.054, 0.061)$	$(0.047, 0.052)$	$(0.052, 0.06)$	$(0.054, 0.061)$
$\mathbb{P}(E_1)$	0.06	0.84	0.04	0.01	0.20
$\mathbb{P}(E_2)$	0.04	0.08	0.01	0.01	0.20
$\mathbb{P}(E_3)$	0.90	0.08	0.95	0.98	0.60
			16,000 cells per well		
PDE Error (%)	$(10.642, 14.6)$	$(8.664, 12.528)$	$(10.425, 15.873)$	$(9.364, 14.321)$	$(8.452, 13.372)$
α_1	—	$(-2.251, -0.001)$	—	$(-2.145, -0.079)$	$(-2.632, -0.455)$
α_2 (h^{-1})	—	$(0.101, 0.334)$	—	$(0.102, 0.347)$	$(0.212, 0.487)$
β_1 (µm^2 h^{-1})	$(383.114, 648.525)$	$(434.754, 728.258)$	—	—	$(381.943, 732.084)$
β_2 (µm^2 h^{-1})	—	—	$(810.515, 1541.17)$	$(781.53, 1527.24)$	$(-623.523, 224.81)$
β_3	—	—	—	—	$(4.547, 7.077)$
γ_1 (h^{-1})	$(0.049, 0.055)$	$(0.056, 0.066)$	$(0.048, 0.054)$	$(0.055, 0.065)$	$(0.052, 0.064)$
$\mathbb{P}(E_1)$	0.01	0.92	0.00	0.07	0.16
$\mathbb{P}(E_2)$	0.00	0.01	0.00	0.00	0.21
$\mathbb{P}(E_3)$	0.99	0.07	1.00	0.93	0.63
			18,000 cells per well		
PDE Error (%)	$(8.232, 11.244)$	$(6.793, 9.209)$	$(8.6, 12.424)$	$(7.125, 10.417)$	$(6.767, 10.259)$
α_1	—	$(-1.496, 0.056)$	—	$(-1.428, -0.001)$	$(-1.169, -0.451)$
α_2 (h^{-1})	—	$(0.059, 0.239)$	—	$(0.054, 0.209)$	$(0.084, 0.19)$
β_1 (µm^2 h^{-1})	$(432.41, 761.145)$	$(470.122, 922.102)$	—	—	$(300.382, 955.09)$
β_2 (µm^2 h^{-1})	—	—	$(667.259, 1591.49)$	$(532.904, 1639.44)$	$(-597.988, 189.716)$
β_3	—	—	—	—	$(4.538, 6.515)$
γ_1 (h^{-1})	$(0.054, 0.061)$	$(0.064, 0.089)$	$(0.053, 0.06)$	$(0.063, 0.092)$	$(0.065, 0.087)$
$\mathbb{P}(E_1)$	0.05	0.79	0.00	0.00	0.32
$\mathbb{P}(E_2)$	0.02	0.06	0.00	0.00	0.08
$\mathbb{P}(E_3)$	0.93	0.15	1.00	1.00	0.60
			20,000 cells per well		
PDE Error (%)	$(8.891, 11.326)$	$(7.07, 9.531)$	$(11.118, 14.583)$	$(8.136, 11.768)$	$(7.153, 10.406)$
α_1	—	$(-4.478, -1.94)$	—	$(-2.452, -1.758)$	$(-1.679, -0.885)$
α_2 (h^{-1})	—	$(0.302, 0.532)$	—	$(0.275, 0.392)$	$(0.152, 0.24)$
β_1 (µm^2 h^{-1})	$(402.265, 663.327)$	$(446.431, 850.315)$	—	—	$(512.724, 12161.19)$
β_2 (µm^2 h^{-1})	—	—	$(646.442, 1202.99)$	$(636.312, 1463.91)$	$(-1095.9, -441.254)$
β_3	—	—	—	—	$(1.156, 1.472)$
γ_1 (h^{-1})	$(0.069, 0.077)$	$(0.083, 0.93)$	$(0.072, 0.084)$	$(0.086, 0.101)$	$(0.082, 0.098)$
$\mathbb{P}(E_1)$	0.00	0.84	0.00	0.00	0.24
$\mathbb{P}(E_2)$	0.00	0.07	0.00	0.00	0.13
$\mathbb{P}(E_3)$	1.00	0.09	1.00	1.00	0.63

Table H: Model selection results for the five models considered with $N_p = 50$. Interval estimates, PDE errors, and model selection results for the models in (2)–(6) when applied to each data set from Jin et al. [1]. See the paper for more details. The time taken to produce these results is 56 minutes and 7 seconds.

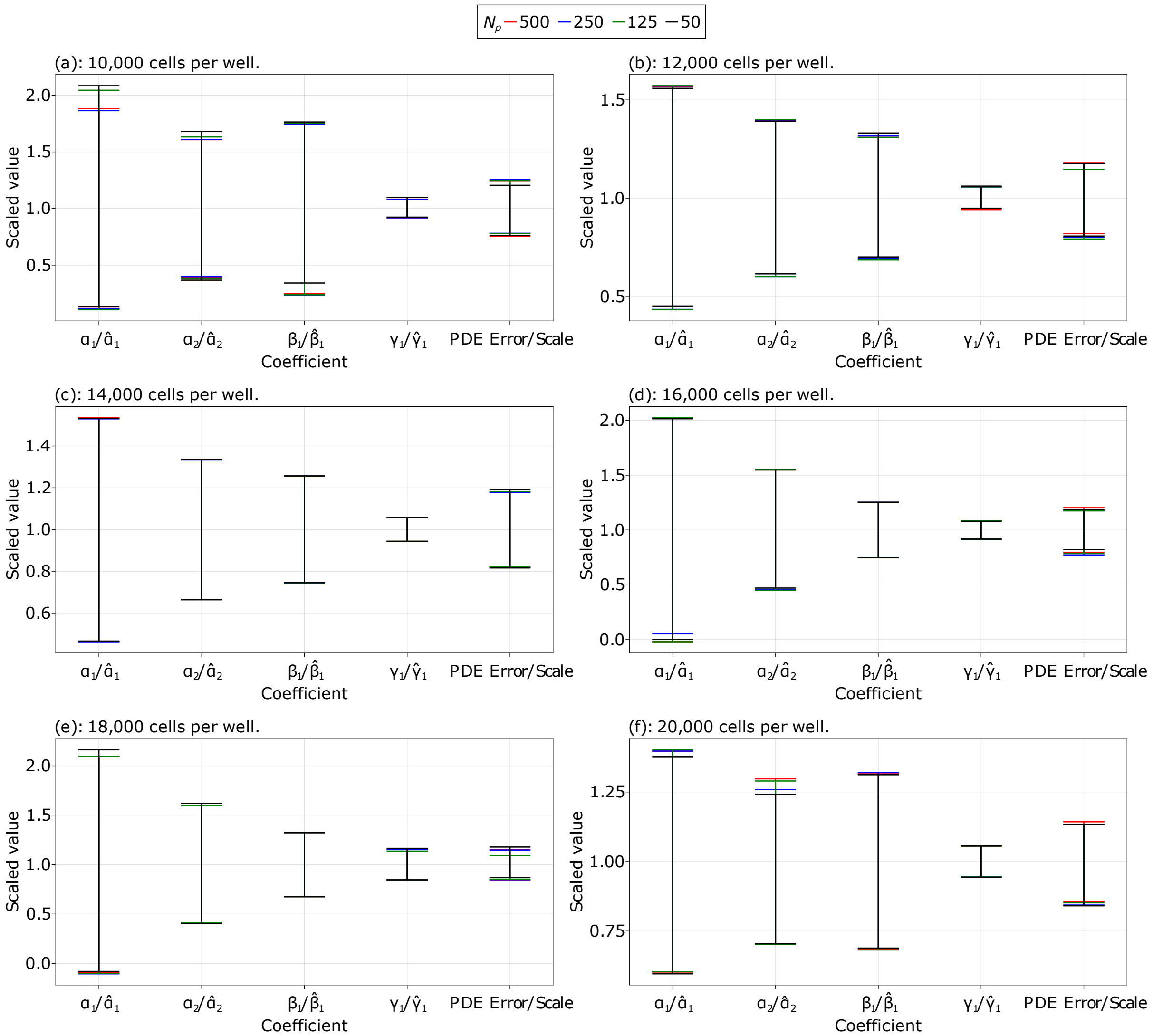

Figure V: Coefficient comparisons for different grid sizes. Comparing coefficients from model 2 for each data set, using different values for N_p. The lines shown are the 95% confidence intervals for the corresponding coefficient. The coefficients are all scaled by a value $\widehat{\theta}$, e.g. α_1 is scaled by $\widehat{\alpha}_1$, where $\widehat{\theta}$ is the mean value of the coefficient from the results using $N_p = 500$ for that data set, and similarly for the PDE errors.

S6 Notation

In this section we present tables of notation used throughout the manuscript.

Notation	Description
$(x_1, \ldots, x_N)$	The spatial grid for the experiment, where $x_1 < \cdots < x_N$. Also used for the PDE grid, although the PDE grid goes up to x_{N_p}.
$(t_1, \ldots, t_M)$	The temporal grid for the experiment, where $t_1 < \cdots < t_M$.
$(x_1^*, \ldots, x_n^*)$	New points placed between $[x_1, x_N]$ used for bootstrapping.
$(t_1^*, \ldots, t_m^*)$	New points placed between $[t_1, t_M]$ used for bootstrapping.
$(\xi_1, \ldots, \xi_n)$	Scaled version of $(x_1^*, \ldots, x_n^*)$ such that the ξ_i are all in $[0, 1]$.
$(\tau_1, \ldots, \tau_m)$	Scaled version of $(t_1^*, \ldots, t_m)$ such that the τ_j are all in $[0, 1]$.
u_{ij}	The experimentally measured density value at (x_i, t_j).
u_{ij}^*	The estimate for the cell density from the Gaussian process..
$\widehat{u}_{ij}$	The estimate for the cell density from the numerical solution of the differential equations, given over the experimental grid (x_i, t_j).
$\widehat{u}_{ij}^{(b)}$	Estimates for the cell densities from the numerical solution of the differential equations corresponding to the bth bootstrap iteration at (x_i, t_j).
N	The number of spatial grid points for the experiment.
M	The number of temporal grid points for the experiment.
n	The number of spatial grid points for the bootstrapping grid.
m	The number of temporal grid points for the bootstrapping grid.
N_p	The number of spatial grid points for the discretisation of the PDE.

Table I: Grid and density notation. Notation used for several grids and for labelling the density values.

References

[1] Jin W, Shah ET, Penington CJ, McCue SW, Chopin LK, Simpson MJ. Reproducibility of scratch assays is affected by the initial degree of confluence: Experiments, modelling and model selection. Journal of Theoretical Biology. 2016; 390:136–145.

[2] Dierckx P. Curve and surface fitting with splines. Oxford: Oxford University Press; 1993.

[3] Barbary K. Dierckx.jl. GitHub Repository. 2014 [cited 2022 May 10]. Available from: https://github.com/kbarbary/Dierckx.jl.

[4] Bezanson J, Edelman A, Karpinski S, Shah VB. Julia: A fresh approach to numerical computing. SIAM Review. 2017; 59:65–98.

[5] Lagergren JH, Nardini JT, Baker RE, Simpson MJ, Flores KB. Biologically-informed neural networks guide mechanistic modeling from sparse experimental data. PLoS Computational Biology. 2020; 16:e1008462.

Notation	Description
$\mathbf{u} \in \mathbb{R}^{NM\times 1}$	The experimental density values, $\mathbf{u} = (u_{11}, u_{21}, \ldots, u_{N1}, u_{12}, \ldots, u_{NM})^{\mathsf{T}}$.
$\mathbf{X} \in \mathbb{R}^{2\times NM}$	The design matrix for the Gaussian process. The first row is $(x_1, \ldots, x_N)$ repeated M times, and the second row is $(t_1, \ldots, t_1, t_2, \ldots, t_M)$, where each t_i is repeated N times followed by the next t_{i+1}, $i = 1, \ldots, M$.
$\mathbf{u}^* \in \mathbb{R}^{nm\times 1}$	A random variable for the cell densities from the Gaussian process, defined such that $\mathbf{u} = \mathbf{u}^* + \sigma_n \mathbf{z}$ or $\mathbf{u}^* \mid \mathbf{X}, \mathbf{u}, \mathbf{X}^* \sim \mathcal{N}(\bar{\mathbf{u}}^*, \mathbb{C}\mathrm{ov}(\mathbf{u}^*))$.
$\mathbf{X}^* \in \mathbb{R}^{2\times nm}$	Similarly to $\mathbf{X}$, except instead of $(x_1, \ldots, x_N)$ we use $(\xi_1, \ldots, \xi_n)$ and for $(t_1, \ldots, t_M)$ we use $(\tau_1, \ldots, \tau_m)$.
$\mathbf{K}(\mathbf{P}, \mathbf{Q})$	A matrix whose (i, j) value gives the value of the kernel function at $\mathbf{p}_i$ and $\mathbf{q}_j$, $\mathbf{K}_{ij} = k(\mathbf{p}_i, \mathbf{q}_j)$.
$k(\mathbf{p}_i, \mathbf{q}_j)$	The squared-exponential kernel, $k(\mathbf{p}_i, \mathbf{q}_j) = \sigma_f^2 \exp\left\{-\frac{1}{2}(\mathbf{p}_i - \mathbf{q}_j)^{\mathsf{T}} \mathbf{\Lambda}^{-1} (\mathbf{p}_i - \mathbf{q}_j)\right\}$, where $\mathbf{\Lambda} = \mathrm{diag}(\ell_1^2, \ell_2^2)$.
$\mathbf{I}_N$	The N-square identity matrix.
$\bar{\mathbf{u}}^* \in \mathbb{R}^{nm\times 1}$	The mean of the Gaussian process, $\bar{\mathbf{u}}^* = \mathbf{K}(\mathbf{X}^*, \mathbf{X}) \left[\mathbf{K}(\mathbf{X}, \mathbf{X}) + \sigma_n^2 \mathbf{I}_{NM}\right]^{-1} \mathbf{u}$.
$\mathbb{C}\mathrm{ov}(\mathbf{u}^*) \in \mathbb{R}^{nm\times nm}$	The covariance matrix of the Gaussian process, $\mathbb{C}\mathrm{ov}(\mathbf{u}^*) = \mathbf{K}(\mathbf{X}^*, \mathbf{X}^*) - \mathbf{K}(\mathbf{X}^*, \mathbf{X}) \left[\mathbf{K}(\mathbf{X}, \mathbf{X}) + \sigma_n^2 \mathbf{I}_{NM}\right]^{-1} \mathbf{K}(\mathbf{X}, \mathbf{X}^*)$.
$\mathbf{z}$	The random variable $\mathbf{z} \sim \mathcal{N}(\mathbf{0}, \mathbf{I})$.
σ_n	The standard deviation of the noise in the data.
σ_f	The standard deviation of the noise-free data.
ℓ_1	The spatial length scale.
ℓ_2	The temporal length scale.
w	The number of optimiser restarts.
$\mathcal{N}(\boldsymbol{\mu}, \mathbf{\Sigma})$	A normal distribution with mean $\boldsymbol{\mu}$ and covariance matrix $\mathbf{\Sigma}$.
$\boldsymbol{\mu}$	The mean of the random variable $(\mathbf{u}^*, \partial\mathbf{u}^*/\partial\tau, \partial\mathbf{u}^*/\partial\xi, \partial^2\mathbf{u}^*/\partial\xi^2) \mid \mathbf{X}, \mathbf{u}, \mathbf{X}^*$.
$\mathbf{\Sigma}$	The covariance matrix of the same random variable above.
$\mathbf{U}$	A random sample, $\mathbf{U} = \boldsymbol{\mu} + \mathbf{L}\mathbf{z}$.
$\mathbf{L}$	The Cholesky decomposition of $\mathbf{\Sigma}$.
$\mathbf{\Sigma}_{ii}$	The diagonal components of $\mathbf{\Sigma}$, $\mathrm{diag}(\mathbf{\Sigma}) = \mathrm{diag}(\mathbf{\Sigma}_{11}, \mathbf{\Sigma}_{22}, \mathbf{\Sigma}_{33}, \mathbf{\Sigma}_{44})$, where each block is of size $nm \times nm$.
η, η_i, $\boldsymbol{\eta}$	Nugget terms, and $\boldsymbol{\eta} = (\eta_1 \mathbf{I}_{nm}, \eta_2 \mathbf{I}_{nm}, \eta_3 \mathbf{I}_{nm}, \eta_4 \mathbf{I}_{nm})$.

Table J: Gaussian process notation. Notation used for the Gaussian processes.

Notation	Description
$T(t;\boldsymbol{\alpha})$	The delay function with parameters $\boldsymbol{\alpha} = (\alpha_1, \ldots, \alpha_a)^\mathsf{T}$.
$D(u;\boldsymbol{\beta})$	The nonlinear diffusivity function with parameters $\boldsymbol{\beta} = (\beta_1, \ldots, \beta_d)^\mathsf{T}$.
$R(u;\boldsymbol{\gamma})$	The reaction function with parameters $\boldsymbol{\gamma} = (\gamma_1, \ldots, \gamma_r)^\mathsf{T}$
K	The measured carrying capacity density from Jin et al. [1], given as $K = 1.7 \times 10^{-3}\,\text{cells}\,\mu\text{m}^{-2}$.
$\boldsymbol{\theta}$	The parameters $\boldsymbol{\theta} = (\boldsymbol{\alpha}^\mathsf{T}, \boldsymbol{\beta}^\mathsf{T}, \boldsymbol{\gamma})^\mathsf{T}$.
$\mathcal{L}_{\text{PDE}}(\boldsymbol{\theta})$	The loss function over the errors in the assumed form the PDE.
$\mathcal{L}_{\text{GLS}}(\boldsymbol{\theta})$	The loss function between the data and the numerical solution of the PDE, using the scaled residuals $(\widehat{u}_{ij} - u_{ij})/\sigma_n$
$\mathcal{L}(\boldsymbol{\theta})$	The loss function $\mathcal{L}(\boldsymbol{\theta}) = \log[\mathcal{L}_{\text{PDE}}(\boldsymbol{\theta})] + \log[\mathcal{L}_{\text{GLS}}(\boldsymbol{\theta})]$.
$\widehat{\boldsymbol{\theta}}$	An estimate for the parameters $\widehat{\boldsymbol{\theta}} = (\widehat{\boldsymbol{\alpha}}^\mathsf{T}, \widehat{\boldsymbol{\beta}}^\mathsf{T}, \widehat{\boldsymbol{\gamma}}^\mathsf{T})^\mathsf{T}$, given as $\widehat{\boldsymbol{\theta}} = \operatorname{argmin}_{\boldsymbol{\theta} \in \mathbb{R}^{a+d+r}} \mathcal{L}(\boldsymbol{\theta})$.
a	The number of delay parameters.
d	The number of diffusion parameters.
r	The number of reaction parameters.
θ_i	The ith parameter in $\boldsymbol{\theta}$.
B	The number of bootstrap iterations.
$\mathbf{z}^{(b)}$	A sample from $\mathcal{N}(\mathbf{0}, \mathbf{I})$.
$\mathbf{U}^{(b)}$	A sample from the joint Gaussian process, $\mathbf{U}^{(b)} = \boldsymbol{\mu} + \mathbf{L}\mathbf{z}^{(b)}$.
AIC	The Akaike information criterion.
AICc	The Akaike information criterion, corrected for a small sample size.
$\lvert\mathcal{M}\rvert$	The number of proposed models.
$\mathcal{M}_i$	The ith candidate model, $i = 1, \ldots, \lvert\mathcal{M}\rvert$.
AICc_i	The AICc of $\mathcal{M}_i$.
AICc_{min}	The minimum AICc over a set of AICcs from a set of candidate models.
Δ_i	The AICc difference, $\Delta_i = \text{AICc}_i - \text{AICc}_{\text{min}}$.
$\mathcal{M}_i^{(b)}$	The bth realisation of the model $\mathcal{M}_i$, where $i = 1, \ldots, \lvert\mathcal{M}\rvert$ and $j = 1, \ldots, B$.
$\text{AICc}_i^{(b)}$	The AICc of $\mathcal{M}_i^{(b)}$.
$\mathbf{P} \in \mathbb{R}^{3 \times \lvert\mathcal{M}\rvert}$	The matrix for probabilistic interpretations for model selection from the AICc results.
ϕ_k	The kth basis function for the nonlinear diffusivity function for the basis function approach.
ψ_k	The kth basis function for the reaction function for the basis function approach.
$\mathbf{A} \in \mathbb{R}^{nm \times (d+r)}$	The coefficient matrix for the system $\mathbf{A}\boldsymbol{\theta} = \partial \mathbf{u}_* / \partial x$ for the basis function approach.
δu	The threshold tolerance for the density values.
δt	The threshold tolerance for the time derivatives of the density values.

Table K: Equation learning, bootstrapping, and model selection notation. Notation used for the equation learning, bootstrapping, and model selection.
