# Supplementary material for: Computationally efficient mechanism discovery for cell invasion with uncertainty quantification
Source: PLoS Comput Biol. 2022 Nov 16;18(11):e1010599. doi: 10.1371/journal.pcbi.1010599 (PMC9710850; doi:10.1371/journal.pcbi.1010599)
Supplement: Attachment — Submitted filename: vandenheuvel2022_review.pdf [file pcbi.1010599.s002.pdf]

1. Computationally efficient mechanism discovery for cell invasion with uncertainty quantification

2. Overview

VandenHeuvel et al. present an approach for estimating both the functional form and parameters of a model to describe cell invasion in scratch-assay experiments. Their approach uses Gaussian processes (GPs) with exponential kernels to fit the mean cell density as a function of space and time. Using GPs confers a number of benefits: that the temporal and spatial derivatives (which are required in their cost functions used to estimate the form of the PDE) are readily obtainable after the GP is fit; and crucially, that the mean cell density and its derivatives are estimated with uncertainty -- this uncertainty is then propagated through to their parameter fitting allowing them to obtain a measure of parameter estimate uncertainty. This estimation step is parallelisable over draws from the fitted GP meaning it avoids the issues inherent with other approaches, such as Markov chain Monte Carlo. The authors apply their method to a number of repetitions and variations of scratch-assay experiments and find that, broadly, a Fisher-Kolmogorov model provides the best fit to the data (as metered by a corrected AIC).

I enjoyed reading this manuscript. It is well-written and the methods sections are comprehensive. The approach that the authors develop is interesting and provides a potentially useful tool for fitting expensive models to data and for quantifying the uncertainty in the fit. With some changes, mainly I suggest to the discussion, I think this work would fit nicely within this journal.

In what follows, I separate my comments into "substantive" and "other" sections.

3. Substantive comments

Overall, I felt that the discussion represented a comprehensive assortment of all things that could be done for fitting this particular scratch-assay dataset rather than providing a broader perspective on the use and usefulness of this type of approach. I recognise that some of these limitations need to be covered, but I think there are potentially more interesting (and useful) things that could be discussed and concluded. For instance, it is clear from the comprehensive methods section that getting this approach to work required much work and tinkering. This, of course, is typical of computational biology and not a problem for the current work. But it does beg the questions: could this method be widely adopted?; and what problems would need to be solved in order for the inference method to be deployed in a more automated fashion? These are pertinent because more (inferentially) expensive methods such as Bayesian inference require much less work to set up the problem and the measure of uncertainty obtained has more theoretical grounding.

Additionally, I would be interested to hear what the authors thought about whether numerical error induced by solving the PDE system at disparate parameter sets (those sets potentially requiring different grid sizes) would affect the inferences obtained.

4. Other comments

4.1. Abstract

- "All code and data to reproduce this work is available" -> "All code and data to reproduce this work are available"

4.2. Author summary

- "where only sparse noisy data is available." -> "where only sparse noisy data are available."

4.3. Introduction

- "Biological data is often sparse" -> "Biological data are often sparse"
- "the data is typically noisy" -> "the data are typically noisy"
- "commonly-used" -> "commonly used" since adverbs ending in "ly" don't need hyphens
- "informed neural network for equation learning, and demonstrate their methods". Here, no comma is necessary since it is a dependent clause
- "Bayesian approaches for model selection are well-established [22–24], these methods often rely". This sentence doesn't quite make grammatical sense currently.

4.4. Results and discussion

- "We then discuss the results for a set of models fit to this data" -> "We then discuss the results for a set of models fit to these data"
- "Images of the experiment at equally spaced time intervals at $t = 0, 12, 24, 36$, and 48 h." -> "Images of the experiment are at equally spaced time intervals at $t = 0, 12, 24, 36$, and 48 h."
- I really liked figure 1: I thought it really brought to life how the scratch assays worked
- Figure 1 caption, "The 1900 μm wide blue rectangle shows the region where data is imaged and analysed" -> "The 1900 μm wide blue rectangle shows the region where data are imaged and analysed"
- Figure 2: the figure titles looked a little low resolution; the figure titles also used 12 / 48 hours opposed to 12 / 48 h in the text
- I don't think there's a need to repeat the numeric value "thus we treat $K = 1.7\times10^{-3}$ cells μm^{-2} as a fixed quantity." since it was only introduced in the previous sentence
- "We see that the data is reasonably noisy around these lines." -> "We see that the data are reasonably noisy around these lines."
- In Figure 4 caption:
    - I think it would be useful to explicitly state what the uncertainty interval represents: for

    instance, if it represents +- a standard deviation or posterior quantiles.
    - "than u, K = 1.7 × 10−3 cells μm−2." -> "than u, where K = 1.7 × 10−3 cells μm−2."
- "It is to these Gaussian processes that we sample from in order to learn the mechanisms driving this invasion." -> "We then sample from these Gaussian processes to learn the mechanisms driving invasion."
- In equation 9, it took me a while to work out that the two epsilons represented different things (the MAPE and machine precision): I'd suggest changing the notation for one of them to make this clear.
- "We could thus suggest that for these datasets the delayed generalised..." -> "This implies that the delayed generalised Porous-FKPP model ... reduces to the delayed Fisher-Kolmogorov model for this dataset"
- "We show this visually in the supplementary material where we see that the nonlinear diffusivity curves for model" -> "We show this visually in the supplementary material which illustrates that the nonlinear diffusivity curves for model..."
- I found this sentence quite hard to parse (mainly because of its length): "These larger densities are typically only met for the data at t = 48 h, or only at the endpoints for earlier times..."
- "It is only for the 20,000 CPW dataset that there would be strong evidence to reject..." -> "Only for the 20,000 CPW dataset was there strong evidence to reject..."
- "in contrast to the nonlinearity diffusivity curves found by Lagergren et al.". Is "nonlinearity" right here or would "nonlinear" work?
- I'm not sure I understand your sentence, "D(u; β) should not be assumed to be monotonic" since above you say that "we note that all the D(u; β) functions are decreasing for this model"?

4.5. Conclusions

- "The data considered in this work is sparse and noisy." -> "The data considered in this work are sparse and noisy."

4.6. Methods

- "Following this discussion we describe how we solve the PDEs numerically." -> "We then describe how we solve the PDEs numerically."
- I wasn't sure what this sentence meant, "the value of w1 = 10 depends on the data...." since it is a fixed value
