# Supplementary material for: Computationally efficient mechanism discovery for cell invasion with uncertainty quantification
Source: PLoS Comput Biol. 2022 Nov 16;18(11):e1010599. doi: 10.1371/journal.pcbi.1010599 (PMC9710850; doi:10.1371/journal.pcbi.1010599)
Supplement: Attachment — Submitted filename: Revision_Letter.pdf [file pcbi.1010599.s003.pdf]

Date August 7, 2022
Contact Matthew J. Simpson
Phone +61 4 1369 6607
E-mail matthew.simpson@qut.edu.au
Subject Manuscript Submission

Professor Matthew J. Simpson

School of Mathematical Sciences
Science and Engineering Faculty
Queensland University of Technology
GPO Box 2434, GP Campus
Brisbane, Queensland 4001 Australia

Philip Maini
Associate Editor
PLOS Computational Biology

Dear Philip,

I am writing to submit a revised manuscript, "Computationally efficient mechanism discovery for cell invasion with uncertainty quantification", that is being considered for publication in the *PLOS Computational Biology*. We appreciate the supportive comments from the three Referees, and we have revised the manuscript to address all comments raised. In summary, to address the comments from Referee 1 (Dr Ben Lambert) we have revised the manuscript to provide more information about how the framework can be applied more generally, and we also added new results in the Supplementary Material document where we systematically explore the impact of truncation error by choosing different discretisation levels when solving the underlying PDE model. To address the comments from Referee 2 we now provide more description about the Gaussian process approach, and we give a more comprehensive discussion about how things change if we wish to estimate the carrying capacity density along with the other parameters, and we also provide more discussion about working with misspecified models. Finally, to address the comments from Referee 3 we now provide point estimates of parameter values, we have clarified some of our terminology, and clarified our claims about the approach being computationally efficient. Of course, in addition to these main points, we have also addressed all of the typographical errors throughout the manuscript.

All changes to the manuscript are highlighted in blue font, and a point-by-point response to each comment is attached. We thank you for your time, and hope that you find the revised manuscript suitable for publication.

Yours sincerely,

Matthew J. Simpson

Referee : Dr Ben Lambert

1. VandenHeuvel et al. present an approach for estimating both the functional form and parameters of a model to describe cell invasion in scratch-assay experiments. Their approach uses Gaussian processes (GPs) with exponential kernels to fit the mean cell density as a function of space and time. Using GPs confers a number of benefits: that the temporal and spatial derivatives (which are required in their cost functions used to estimate the form of the PDE) are readily obtainable after the GP is fit; and crucially, that the mean cell density and its derivatives are estimated with uncertainty – this uncertainty is then propagated through to their parameter fitting allowing them to obtain a measure of parameter estimate uncertainty. This estimation step is parallelisable over draws from the fitted GP meaning it avoids the issues inherent with other approaches, such as Markov chain Monte Carlo. The authors apply their method to a number of repetitions and variations of scratch-assay experiments and find that, broadly, a Fisher-Kolmogorov model provides the best fit to the data (as metered by a corrected AIC). I enjoyed reading this manuscript. It is well-written and the methods sections are comprehensive. The approach that the authors develop is interesting and provides a potentially useful tool for fitting expensive models to data and for quantifying the uncertainty in the fit. With some changes, mainly I suggest to the discussion, I think this work would fit nicely within this journal.

   Response: We thank Dr Lambert for his report, and we now address his questions.

2. Overall, I felt that the discussion represented a comprehensive assortment of all things that could be done for fitting this particular scratch-assay dataset rather than providing a broader perspective on the use and usefulness of this type of approach. I recognise that some of these limitations need to be covered, but I think there are potentially more interesting (and useful) things that could be discussed and concluded. For instance, it is clear from the comprehensive methods section that getting this approach to work required much work and tinkering. This, of course, is typical of computational biology and not a problem for the current work. But it does beg the questions: could this method be widely adopted?; and what problems would need to be solved in order for the inference method to be deployed in a more automated fashion? These are pertinent because more (inferentially) expensive methods such as Bayesian inference require much less work to set up the problem and the measure of uncertainty obtained has more theoretical grounding.

   Response: We agree that this is an important consideration. We believe our method applies to other partial differential equation models for different applications beyond collective cell migration and proliferation. The main change a user would have to make is to the Gaussian process itself, by choosing an appropriate kernel and noise model for the application and data of interest. Our approach is always to work with the most straightforward approach in the first instance which is why we work with the simple squared exponential model with a constant noise level, but different applications could require more sophisticated kernels and data-dependent noise. Beyond these considerations, little else would need configuring. This feature is appealing relative to more standard Bayesian methods which may require significantly more time spent thinking about and experimenting with different priors, sampling schemes, and tuning parameters of the sampling scheme. We have added a brief discussion to the end of the conclusion to make this clear (end of Page 21–22).

3. Additionally, I would be interested to hear what the authors thought about whether numerical error induced by solving the PDE system at disparate parameter sets (those sets

potentially requiring different grid sizes) would affect the inferences obtained.

Response: This is an interesting question. While the temporal discretisation is chosen adaptively by the Julia software that we have developed and released, the spatial discretisation could be important. We currently use 500 equally-spaced gridpoints throughout. To address this question we added a new section to the supplementary material (Supplementary Material Section S5, Pages S24–S30) where we systematically explore working with 250, 125, and 50 equally-spaced spatial gridpoints. We find that these differences do not change any of the results or conclusions, and in fact the 50 gridpoint case allows all results in the manuscript to be generated in under one hour on a single laptop machine, compared to the 30 hour run-time with 500 spatial gridpoints. Hence, we find that the truncation error induced by solving the PDE system at different parameter sets does not dramatically affect the inferences obtained for this problem. We also summarise these results in the revised manuscript (end of Page 31–32).

4. "All code and data to reproduce this work is available" $\rightarrow$ "All code and data to reproduce this work are available"

   Response: We agree, and made this change (Page 1, Paragraph 1).

5. "where only sparse noisy data is available." $\rightarrow$ "where only sparse noisy data are available."

   Response: We agree, and made this change (Page 1, Paragraph 2).

6. "Biological data is often sparse" $\rightarrow$ "Biological data are often sparse"

   Response: We agree, and made this change (Page 2, Paragraph 2).

7. "the data is typically noisy" $\rightarrow$ "the data are typically noisy"

   Response: We agree, and made this change (Page 2, Paragraph 2).

8. "commonly-used" $\rightarrow$ "commonly used" since adverbs ending in "ly" don't need hyphens

   Response: We agree, and made this change (Page 2, Paragraph 2).

9. "informed neural network for equation learning, and demonstrate their methods". Here, no comma is necessary since it is a dependent clause

   Response: We agree, and made this change (Page 3, Paragraph 1).

10. "Bayesian approaches for model selection are well-established [22–24], these methods often rely". This sentence doesn't quite make grammatical sense currently.

    Response: We agree, and have now updated this sentence to be clearer (Page 3, Paragraph 3).

11. "We then discuss the results for a set of models fit to this data" $\rightarrow$ "We then discuss the results for a set of models fit to these data"

    Response: We agree, and made this change (Page 4, Paragraph 4).

12. "Images of the experiment at equally spaced time intervals at t = 0, 12, 24, 36, and 48 h." $\rightarrow$ "Images of the experiment are at equally spaced time intervals at t = 0, 12, 24, 36, and 48 h."

    Response: We agree, and made this change (Page 5, Paragraph 2).

13. I really liked figure 1: I thought it really brought to life how the scratch assays worked

    Response: We appreciate this comment, thank you.

14. Figure 1 caption, "The 1900 μm wide blue rectangle shows the region where data is imaged and analysed" $\rightarrow$ "The 1900 μm wide blue rectangle shows the region where data are imaged and analysed"

    Response: We agree, and made this change (Page 6, Figure 1 Caption).

15. Figure 2: the figure titles looked a little low resolution; the figure titles also used 12 / 48 hours opposed to 12 / 48 h in the text

    Response: We agree, and have now updated the figure resolution and changed the units to be consistent (Page 7, Figure 2).

16. I don't think there's a need to repeat the numeric value "thus we treat $K = 1.7 \times 10^{-3}$ cells μm $^{-2}$ as a fixed quantity." since it was only introduced in the previous sentence

    Response: We agree, and made this change (Page 6, Paragraph 2).

17. "We see that the data is reasonably noisy around these lines." $\rightarrow$ "We see that the data are reasonably noisy around these lines."

    Response: We agree, and made this change (Page 7, Paragraph 2).

18. In Figure 4 caption, I think it would be useful to explicitly state what the uncertainty interval represents: for instance, if it represents +- a standard deviation or posterior quantiles.

    Response: We agree, and updated the figure caption to note that the uncertainty interval is a confidence interval of the form $\hat{\mu} \pm 1.96\,\mathrm{se}$, where $\hat{\mu}$ is the mean at the point and se is the standard error at the point (Page 9, Figure 4 Caption).

19. In Figure caption, "than u, $K = 1.7 \times 10^{-3}$ cells μm $^{-2}$." $\rightarrow$ "than u, where $K = 1.7 \times 10^{-3}$ cells μm $^{-2}$."

    Response: We agree, and made this change (Page 9, Figure 4 Caption).

20. "It is to these Gaussian processes that we sample from in order to learn the mechanisms driving this invasion." $\rightarrow$ "We then sample from these Gaussian processes to learn the mechanisms driving invasion."

    Response: We agree with this suggestion, although this paragraph now has additional changes (See Response 4 to Referee 3 where we have updated Pages 8–10 of Section 3.2).

21. In equation 9, it took me a while to work out that the two epsilons represented different things (the MAPE and machine precision): I'd suggest changing the notation for one of them to make this clear.

    Response: We agree that this can be unclear, and have changed the notation for the machine precision to be ζ rather than ε (Page 14, Equation 9 and Paragraph 1).

22. "We could thus suggest that for these datasets the delayed generalised..." $\rightarrow$ "This implies that the delayed generalised Porous-FKPP model ... reduces to the delayed Fisher- Kolmogorov model for this dataset".

    Response: We agree, and made this change (Page 15, Paragraph 2).

23. "We show this visually in the supplementary material where we see that the nonlinear diffusivity curves for model" $\rightarrow$ "We show this visually in the supplementary material which illustrates that the nonlinear diffusivity curves for model..."

    Response: We agree, and made this change (Page 15, Paragraph 2).

24. I found this sentence quite hard to parse (mainly because of its length): “These larger densities are typically only met for the data at t = 48 h, or only at the endpoints for earlier times...”

    Response: We agree that the length of this sentence makes it difficult to comprehend. We have now split this sentence into three clearer parts (Page 15, Paragraph 2).

25. “It is only for the 20,000 CPW dataset that there would be strong evidence to reject...” $\rightarrow$ “Only for the 20,000 CPW dataset was there strong evidence to reject...”

    Response: We agree, and have made this change (Page 15, Paragraph 2).

26. “in contrast to the nonlinearity diffusivity curves found by Lagergren et al.”. Is “nonlinearity” right here or would “nonlinear” work?

    Response: “Nonlinearity” is incorrect here, so we have changed it to “nonlinear” as suggested (Page 17, Paragraph 2).

27. I’m not sure I understand your sentence, “D(u; β) should not be assumed to be monotonic” since above you say that “we note that all the D(u; β) functions are decreasing for this model”?

    Response: The point we were trying to make is that $D(u; \boldsymbol{\beta})$ should be allowed to be quite general, as opposed to being strictly monotonic as in the work of Lagergren et al. (2020). We have made this clearer by adding a final comment to this sentence commenting that monotonicity can simply be a special case when optimising $D(u; \boldsymbol{\beta})$ rather than the only case (Page 17, Paragraph 2).

28. “The data considered in this work is sparse and noisy.” $\rightarrow$ “The data considered in this work are sparse and noisy.”

    Response: We agree, and made this change (Page 19, Paragraph 2).

29. “Following this discussion we describe how we solve the PDEs numerically.” $\rightarrow$ “We then describe how we solve the PDEs numerically.”

    Response: We agree, and made this change (Page 22, Paragraph 2).

30. I wasn’t sure what this sentence meant, “the value of w1 = 10 depends on the data....” since it is a fixed value.

    Response: We agree this sentence is unclear. The point here was to state that choosing $w_1 = 10$ works well for this dataset, but it could change for other datasets. We have changed this sentence to now be clearer with this point, emphasising that w_1 is simply a hyperparameter (Page 28, Paragraph 1).

Referee 2

1. This study proposes a framework for discovering biological mechanisms using equation learning, with the additional benefit of providing uncertainty quantification estimates. It specifically applies this framework to PDE models of cell invasion scratch assay experiments, where they perform model selection to study mechanisms such as delay, migration, and proliferation. The framework is based on using Gaussian processes combined with bootstrapping, as opposed to neural networks or Bayesian methods previously employed for this type of learning. The method seems to have some advantages in terms of dealing with noisy and sparse data, being parallelizable, and enabling uncertainty quantification. The manuscript is largely well-written, and I recommend the following revisions:

   Response: We thank Referee 2 for their positive report, and we now address their questions.

2. In section 3.1, the authors discuss their procedure for choosing a carrying capacity K for their study. In the Discussion, they briefly mention adding K to the list of parameters to be estimated. Do you have any intuition on how adding such a parameter may make the framework more computationally extensive, or less robust?

   Response: We anticipate our framework will be more computationally expensive if K is to be estimated due to the increased dimension of the parameter space and because K appears in both the $D(u;\boldsymbol{\beta})$ and $R(u;\boldsymbol{\gamma})$ functions. We expect that the difference in performance would not be so large, however, as K is well-identified by the late-time experimental data, meaning that our framework would remain just as robust. To make this point clear, we have added a brief comment regarding this note to the discussion (Page 19, Paragraph 1).

3. This may be discussed in the cited publications, but what is the intuition behind the delay term for these experiments?

   Response: The motivation for the delay term comes from separate experimental evidence provided by Jin et al. (2017) who studied a similar experiment and showed that the physical act of scratching the monolayer of cells leads to a delay in normal cell function, which we now explicitly mention (Page 11, Paragraph 2).

4. Section 3.3.1 could provide a bit more background on the synthetic data studies in the supplement, especially since they find that an iterative approach to parameter re-scaling may be necessary.

   Response: We agree, and have provided more detail in the revised manuscript about how one might use our framework to identify model misspecification (Page 13, Paragraph 1 of Section 3.3.1), and a remark concerning the iterative approach (Page 13, Paragraph 2). We have also added a new paragraph in the supplementary material to clarify how model misspecification can be identified (Pages S7–S8).

5. In section 3.3.2, the authors start describing some ways to quantify error based on bootstraps sampled from the Gaussian process. This becomes clearer once one reads the Methods, but I wonder if moving up some of the Methods or providing an overview of the framework before these sections would make these Results sections more accessible.

   Response: We agree, and have added a short preamble about the methods before presenting the results (Page 12, Paragraph 1).

6. In the long paragraph on page 13, it would be helpful to break down several of the sentences into shorter ones to make the discussion of the hypothesis testing and differences between datasets more readable.

   Response: We agree, and simplified many sentences in this paragraph (Pages 15–17).

7. In the second paragraph on page 13, it was unclear why the previous study (Lagergren et al.) constrained the diffusivity function to be increasing. If there is a biological motivation for this, wouldn't we expect to see the same trend with this new framework?

   Response: We (Simpson) were part of the Lagergren et al. (2020) study, and we confirm that there was no particular biological motivation to constrain the nonlinear diffusivity function to be increasing in that work. This previous choice was simply made for convenience since visual inspection of the experimental images suggest that higher initial densities lead to enhanced wound closure rates. We have added a brief note on this in this same section (Page 17, Paragraph 2).

8. Are there parameter identifiability issues with any of the models considered here? How does this framework connect to results discussed in the authors' 2020 study on "Practical parameter identifiability for spatio-temporal models of cell invasion" for the same datasets?

   Response: We thank the referee for this interesting question. With our approach, parameter identifiability can be informally assessed by examining the kernel density estimates for the parameter estimates obtained using bootstrapping. Densities with especially flat modes would imply practical non-identifiability for that parameter. We have now added a short sentence to describe this (end of Page 20). For the models that we consider, there are no parameter identifiability issues. The main connection with Simpson et al. (2020) is that the method of assessing the flatness of the kernel density estimates is similar to assessing the flatness of a profile likelihood.

9. Does the framework extend to learning biological mechanisms in systems of partial differential equations models?

   Response: We believe the answer to this interesting question is yes. The framework is quite general, with the only real modification required for particular applications is the Gaussian process. For the case of a system of partial differential equations, a Gaussian process with multiple response variables is necessary. We now describe this in the revised discussion (end of Page 21–22).

10. In eq. (14), the authors could extend the second row of matrix X to make it clearer that t_1 is repeated N times, then t_2, etc.

    Response: We agree, and have now extended $\boldsymbol{X}$ to make this clear (Page 23, Equation 14).

11. On page 20 before eq. (17), subscript notation for u_* is used. Is this intended to be the same notation as in eq. (15)? This could be made clearer.

    Response: This $\boldsymbol{u}_*$ was intended to be $\boldsymbol{u}^*$, like in Eq. (15), and has now been corrected (Page 24, immediately before Equation 17).

12. In section 4.3, are there numerical concerns that matrix sigma is not symmetric positive definite?

    Response: There is no concern about the covariance matrix not being symmetric positive definite. This is a common issue when building covariance matrices, and is indeed

related to numerical issues, as discussed on page 201 of "Gaussian Processes for Machine Learning" by Rasmussen and Williams (2006). In particular, the eigenvalues of our covariance matrix decay rapidly. The terms that we have to add to the matrix to force it to be symmetric positive definite, and hence ensuring numerical stability, do not have a significant impact on the resulting analyses as they are equivalent to adding an independent noise term with variance equal to our nugget terms, in this case 10^{-5}. We now briefly comment on this being a common issue (Page 26, Paragraph 2).

13. The authors could provide more intuition on the two loss functions introduced on page 23. For instance, how does the first loss function, which is based on the methodology proposed here (draws from the fitted Gaussian process), improve the optimization?

    Response: The first loss function is used to ensure that the profiles we obtain satisfy the governing PDE. Additionally, as this loss function is where the parameters $\boldsymbol{\theta}$ appear directly, this loss function is the main contributor to the learned uncertainty from our results. The second loss function, $\mathcal{L}_{\text{GLS}}$, is used to guarantee that the profiles do not diverge too far from the experimental data. Hence, $\mathcal{L}_{\text{GLS}}$ considers the sampled values, while $\mathcal{L}_{\text{PDE}}$ considers the experimental data. We have added a note about this following the introduction of these two loss functions (Page 28, Paragraph 1).

14. On top of page 29, "jth sample" should probably be "ith" sample.

    Response: It indeed should have been "ith" sample, and we have made this change (Page 34, Paragraph 2). We sincerely thank Referee 2 for their very careful reading of the manuscript.

15. In the example on page 29, should j=2 to correspond to the second interpretation for the AIC score differences?

    Response: Correct, we have now fixed the example to read $[\boldsymbol{P}]_{i2}$ rather than $[\boldsymbol{P}]_{ij}$ (Page 34, Paragraph 2).

16. Page 4, sentence starting with "Images of the experiment" needs a verb.

    Response: We agree, and have made the change (Page 5, Paragraph 2).

17. Page 5, replace "are showing" with "are shown".

    Response: We agree, and have made the change (Page 5, Paragraph 2).

18. Page 22, typo "Cholesky factory".

    Response: We have corrected this typographical error (Page 26, Paragraph 2).

19. The use of "say" on page 24 seems a bit informal and vague.

    Response: We agree, and have now clarified what is meant by the values here (Page 29, Paragraph 2).

20. Page 28, typo "the its AICc".

    Response: We have now made this change (Page 33, Paragraph 5).

Referee 3

1. The manuscript considers a specific application of quite a generic task: how to select, amongst a set of candidate models, which one is best supported by the data. It adopts a bootstrap (simulation-based) approach, aimed at attributing a notion of uncertainty to the inferred model parameters and selected models. Much is nicely written, though I frequently found myself struggling to follow the narrative and the big picture. There is extensive details about *what* was done in terms of methodological choices, but little explanation/justification about *why* these choices were made and how they impact the statistical properties of the inference. Consequently, it is tricky to follow how the contribution relates to the literature and whether the statistical properties of the approach make it attractive.

   Response: We thank Referee 3 for their report, and we now address their questions.

2. The paper omits to consider classical approaches to model selection. S2 cites recent "Mechanism discovery"/ "equation learning" papers and mentions these, and more classical Bayesian approaches, as alternative methodological approaches to the ones being presented. I do not understand, though, why classical approaches to model selection, such as the likelihood-ratio test (LRT, for nested models) and information-criteria based methods (for not necessarily nested models) [ref 62] are not mentioned here and used for comparison throughout the manuscript. These methods date back decades and seem closest in spirit to the approach being taken here. Note that although the LRT is restricted to comparisons of nested models, it is a method of model selection that incorporates uncertainty quantification, and it is very cheap (requiring only two evaluations of the likelihood).

   Response: We agree these classical approaches are important and should be acknowledged, and have now commented on them in the revised introduction (Page 4, Paragraph 2).

3. "Uncertainty quantification" is emphasised throughout as a key contribution of the approach, without explanation of what this means. "Uncertainty" is an overloaded phrase in statistics, and the notion of uncertainty (and the underlying probability, whether frequentist or subjective) is only useful if it is precisely prescribed what is meant by it. This is not mere statistical fussiness: it is central to understanding the role of GPs in the method in this manuscript, and what the resulting, and currently ambiguous, "uncertainty intervals" and "goodness of fit probabilities" actually mean. I *think* (though I'm not sure) that "uncertainty intervals" for fitted parameters are just classical "confidence intervals" interpreted in the frequentist sense. The "goodness of fit" probabilities are more non-standard (something I'll come back to below)

   Response: We agree it is clearer to refer to "confidence intervals" rather than "uncertainty intervals" which has a less precise meaning, and we have made this change throughout the document. Regarding how to best make clear what "uncertainty quantification" means, we address this in responses 4, 5, and 13 (below).

4. GPs are central to the approach, but the role they play isn't fully explained. My understanding (with apologies if I have misunderstood) is that the approach and rationale is as follows:

   1. The GP is fitted to the experimental data and then simulated from.
   2. The GP (sampled at discrete intervals mimicking experimental resolution, possibly plus additive noise) is being considered as a surrogate for the data-generating process.

3. Therefore, bootstrapping (repeatedly simulating a data set from the GP model) mimics repeated collection of novel data sets.
4. For each bootstrap realisation of the data set, multiple candidate models are fitted. This provides point estimates of model parameters, and rankings of the suitability of candidate models (according to some chosen criterion).
5. Repeating over multiple bootstrap realisations provides a sampling distribution for the parameter estimates and model rankings. These are interpreted in the frequentist sense, and hence can be used to compute confidence intervals.

If this interpretation is correct then (i) I think the manuscript would benefit from spelling out such a rationale clearly, (ii) this makes precise that the "uncertainty intervals" in the results really are just frequentist confidence intervals, and should be called that, (iii) it makes clear that point 2 is absolutely crucial: the entire notion of uncertainty, and reliability of inference, hinges on the GP being a reasonable surrogate for the data-generating process. Too much (resp. too little) uncertainty on the simulated data means too much (resp. too little) uncertainty in the inferred results. If the GP over- or under-smooths then there could be all kinds of undesirable consequences for the model inference (which after all is geared here towards selecting different physical models for "smoothing" the observable variable).

Response: Referee 3 makes a very good point, and their description of how Gaussian processes are used in our work is entirely correct. We agree that making these points clear is important for the work to be easily interpretable and understood by others. Additionally, as mentioned in our response to point 3 (above), we agree that these points make it clear that the uncertainty intervals should be called confidence intervals. We have added a discussion in the Gaussian process section (Section 3.2, Pages 8–10) that clearly describes the procedure, as kindly outlined by Referee 3, regarding how Gaussian processes are used, along with a description of how the uncertainty in the Gaussian process and its smoothness are crucial for providing reliable uncertainty quantification and inference. However, as we discussed further in response to point 5 (below), we describe the Gaussian process instead as a surrogate for the dynamical system describing the denoised data-generating process, noting that we aim to discover the mechanisms driving the cell dynamics rather than reproduce the noisy data itself.

5. Is the data-generating process really being carefully mimicked by the GP? On p6, "the uncertainty intervals do not precisely capture the data points...If we were to capture the data itself, an extra term could be added to the variance estimates" makes it sound as though the authors have in mind a model such as $y_i = f(x_i) + \epsilon_i$ and they are simulating E(y_i) (with some variability between GP realisation, though it is unclear to me how this variability is interpreted) rather than y_i, which would be the natural choice if my interpretation 1-5 above were accurate. Is it even necessary to use GP at all? A common bootstrap approach in a setting like this is to "bootstrap the residuals", i.e., compute a non-parametric estimate $\hat{f}$ of f, compute the residuals $r_i = \hat{f}(x_i) - y_i$, then simulate bootstrapped data as $y_i^* = \hat{f}(x_i) + r_i^*$ where each r_i^* is sampled uniformly at random from the set $\{r_i\}$. This feels a bit more direct, and helps to make sure the variability in the simulated/bootstrap data is comparable to the variability in the experiment data - but are there reasons why a GP is preferable to this?

   Response: We do not attempt to mimic the precise data-generating process with our Gaussian process. Instead, we use the Gaussian process as a surrogate for the dynamical system that best describes the denoised data-generating process, i.e. a surrogate for the same E(y_i) given by Referee 3. To meet this aim we use a Gaussian process rather than, for example, the approach given for bootstrapping the residual. We have

now addressed and clarified this important issue (Page 10, Paragraph 1).

6. "Computational efficiency" is another property of the approach that is emphasised, e.g. in the title but not properly explained. This needs to be carefully spelled out: in various methods including the one you propose, which steps are computationally costly and how many times must they be performed? How does the proposed method sidestep, or require fewer of, the costly steps?

   Response: Qualitatively comparing the main features of our approach with more standard Bayesian approaches suggests that there are many features of our approach that are computationally cheaper. Bayesian approaches require repeatedly solving the PDEs and using a sampling scheme, typically limited to serial sampling, and these steps must be performed for as many samples as required, with potentially some computational waste required for burn-in. In contrast, while our approach still requires repeated numerical solutions of the PDE model, sampling from a Gaussian process is extremely fast and optimising for the parameters across each bootstrap realisation is very cheap compared to sampling, for example, with an MCMC algorithm. These samples only have to be obtained for B bootstrap realisations without any burn-in waste, which could even take under an hour for five models on a single laptop machine, as we report in the new Section S5 of the supplementary material and referenced in the revised manuscript (end of Page 31–32). We also note that our approach could be implemented even more efficiently had we used parallel computing. We have provided some small descriptions in the methods to outline the computational cost of some parts of our method (Page 24, Paragraph 2), and briefly summarised this discussion in (Section 3.4, Page 18, Paragraph 1), noting that some of these points are already briefly mentioned in the introduction.

7. p1 What does "efficient" mean?

   Response: Efficient refers to the computational work required for uncertainty quantification and making inference. We have now mentioned this in the introduction (Page 2, Paragraph 2) to make this clear.

8. On p2 it is unclear to me what the authors mean by "Identifying appropriate terms in a mathematical model is traditionally accomplished through empirical reasoning. Model discovery methods instead ... ". It feels as though "empirical reasoning" encompasses pretty much everything. What are the traditional approaches? Aren't the cited "model discovery methods" just a type of empirical reasoning? Mention here the likelihood-ratio test and IC-based model selection approaches, and how the work in the manuscript relates to them.

   Response: While most of these approaches make use of data, the main distinction between our cases and "empirical reasoning" is that we are far more guided by the results of our algorithm than relying on visual inspection of the data alone. A good example of this is given by Sherratt and Murray (1990), whose important landmark study compare the shapes of PDE-based density profiles with data describing the shrinking shape of an epidermal wound. Importantly, Sherratt and Murray (1990) do not work with experimental estimates of cell densities like we do because the experimental system in their study did not enable them to measure how cell density varies with space and time like Jin et al. did. This example is what we consider as "empirical reasoning", and so we would not consider the cited methods as also being a result of empirical reasoning. Regarding the LRT and IC-based model approaches, they have been added in response to Response 2 (above) (Page 4, Paragraph 2).

9. p2 “realistic models are obtained” needs clarification - in what sense “realistic”? (Aren’t all models wrong?)

   Response: We agree that this can be clarified, and have replaced this comment to be about the terms required in the model than referring to a ”realistic” model (Page 2, Paragraph 3).

10. p3 top: the role of the GP needs explanation here. Why choose a GP, and *how* does it enable UQ?

    Response: We have now added a comment to this part, clarifying that the Gaussian process provides a sampling distribution via repeated sampling, noting that the Gaussian process acts as a surrogate for the underlying dynamical system (Page 3, Paragraph 2; see also Section 3.2, Paragraph 1, Page 8).

11. p3 “sparse data” in what sense? (Compared to many biological data sets, the spatial and temporal resolution seem to be very high here)

    Response: Sparse data refers to having very few data points. In this context, we are referring to the fact that most of these other studies consider synthetic data alone, or they work with different experimental systems where more data can be obtained. We have now commented on this in the same section (Page 3, Paragraph 2), making clear that the main point we are making is that we are using experimental data with a limited number of data points.

12. p3 “Bayesian methods” - needs more explanation. (I’m guessing this refers to methods involving statistical model parameterised in terms of the solution of a DE involving parameters, in which inference is based on the likelihood function of this statistical model - and even if so, introducing the idea here would be helpful for later)

    Response: We have now briefly explained what a Bayesian approach involves (Page 3, Paragraph 3).

13. p3 “[compared with the Bayesian approach] our approach ... allows us to enable a candidate library of terms in the PDE model similar to [equation learning]”. But the number of models considered (p9) is only very small. Why couldn’t a similar number be compared in the Bayesian approach? Isn’t the compute time per evaluation of the likelihood essentially the same, because both entail solving the PDE (presumably the slow bit)? Or is the idea here (and of “computational efficiency” throughout) just that the likelihood needs to be evaluated fewer times, i.e. the # of bootstrap samples is fewer than the number of MCMC iterations?

    Response: The idea here is, indeed, that the likelihood function will be evaluated fewer times than would be required in a Bayesian approach. We have now commented on this specifically in this paragraph (Page 4, Paragraph 1).

14. p4 What did Jin et al do to analyse these data? What was their approach and their conclusions? How do these relate to the approach and conclusion of the current manuscript?

    Response: Jin et al. consider a Fisher-Kolmogorov and a Porous-Fisher model, making use of least squares estimation to estimate the parameters (with K fixed), and find weak evidence that a Porous-Fisher model without delay outperforms a Fisher-Kolmogorov model without delay. Our approach relates to this by extending these ideas and considering a broader family of models together with a more appropriate loss function to match both the PDE and the experimental data. We have added a brief note in (Section 3.1, Page 7) addressing this relationship and what Jin et al. did with the data. Furthermore,

we have added several parts throughout the text to relate the work of Jin et al. back to ours, and also to complete the story of how our work relates to the important previous work of Sherratt and Murray (1990) (end of Page 4–5; Page 19, Paragraph 1).

15. p6 Need explanation of *why* the GP is being introduced. What role does it play, and why that choice? What precisely does the "uncertainty interval" mean?

    Response: We have now added a short sentence at the start of Section 3.2, explaining that the Gaussian process enables uncertainty quantification (Section 3.2, Page 8). Our responses to points 3, 4 and 5 (above) together with this new sentence make it clear why we use a Gaussian process, and we now define what we mean by a confidence interval here and throughout.

16. p8 at this point, it is unclear how the GP facilitates "learning the mechanisms", what you will do with the repeatedly sampled values and the GP derivatives, and what is the optimisation problem that's being referring to.

    Response: To make this point clear we now explicitly mention the loss function used in the optimisation problem, making use of the sampled values and its derivatives (Page 10, Paragraph 1). In terms of how the Gaussian process facilitates learning the mechanisms, this is now made clear from modifications from Referee 3's other responses, e.g. Response 5.

17. p9, eqn (2) - (6): no need to repeat the eqn for R if it is identical in each case.

    Response: We agree, and have now removed the repeated R equations and placed a single mention of it after the model descriptions (Page 12, Equations 2–6 and immediately after Equation 6).

18. p9 eqn (4), (5), i.e. models 3 and 4: it would seem more natural to me to relabel that beta$_1$ as beta$_2$. Then it is clear that the "beta" in models 3 and 4 plays a different role to the beta$_1$ in models 1 and 2, and it makes the model nesting clearer - for example, that model 4 is a special case of model 5.

    Response: We agree. We have now updated these models to use β_2 rather than β_1 for this coefficient, and have updated the text and all relevant tables accordingly (Pages 12–18, Section 3.3). We have also done the same for the same models in the supplementary material (Pages S6–S8, Section S1.2).

19. p9 "We will fit these models to each dataset" needs at least brief explanation here (not just a reference to the methods) especially as the fitting criterion is quite non-standard. Why choose such an unusual loss function (defined later on p23), in terms of the derivative of the state variable, rather than in terms of the state variable itself?

    Response: We agree this can be clearer, and it is has been made clear now in response to Response 5 to Referee 2 (above). For the loss function, the derivatives are needed to constrain the learned solutions to match the dynamical system rather than just the experimental data, and so we are required to have a joint loss for both the data and the learned PDE solutions. Some additional text has also been added after the introduction of these two loss functions that makes this clearer, added in response to Response 13 from Referee 2 (Page 28, immediately after Equation 34).

20. p9 "... then compare the using Akaike IC." What is the implied statistical model associated with the unusual loss function? Doesn't the interpretation of the IC depend on the "likelihood" component of the IC relating to an assumed statistical model, rather than an arbitrarily chosen loss?

Response: We do not rely on a specific statistical model associated with the entire loss function, which we argue is not arbitrary. While the GLS loss function is derived from the same statistical model used for the Gaussian process, namely $u_{ij} = u(x_i, t_j) + \sigma_n z_{ij}$, $z_{ij} \sim \mathcal{N}(0, 1)$, the PDE loss function is required to fit to the underlying dynamical system rather than strictly to the experimental data; these loss functions come from Lagergren et al. (2020). For the interpretation of the information criterion, again following Lagergren et al. (2020) and their use of the information criterion from Banks and Joyner (2017), the statistical model is given by $u_{ij} = u(x_i, t_j) + \sigma_n z_{ij}$, since the AIC is still directly computed according to the experimental data and the numerical solutions, i.e. with the same data as used in the GLS loss function. We have now commented on this following our description of the AIC (end Page 34–35).

21. p11 Probabilities P(E1), P(E2), P(E3). “Prob that the model is in the class of best-fitting models”, “that there is only some evidence”, “that there is no evidence” - again can’t be understood unless the reader jumps forwards to the methods. In what statistical sense are these probabilities to be interpreted?

    Response: These probabilities should be interpreted in a Bayesian sense, with “evidence” in this context being the proportion of belief that we assign to the model being the best approximating model for the cell dynamics. We have now briefly mentioned this alongside this sentence to make this clear (Page 14, Paragraph 2).

22. p12 Table 1 - it’s a bit confusing to have the label “Models” then column labels (2)-(6) which refers to the equation numbers not the model numbers (which differ by 1).

    Response: We agree, and have now updated these headings to give the model numbers rather than the equation numbers (Page 16, Table 1), and similarly for other tables like this in the supplementary material (Tables S1–S2, Tables S5–S8).

23. p12 Table 1 - It would be helpful to have some kind of point estimate for the parameter values, e.g. a modal value from the sampling distributions. An important comparator to include parameter estimates computed as maximum likelihood estimates (MLEs) from the data themselves, rather than the GP surrogate (since this would be a conventional starting point most would take when faced with a data such as this, and a model to fit to the data). Would the point estimates using bootstrap sampling be close to the MLE? One would hope that the MLEs would lie well inside the “uncertainty intervals” presented in this table, but there is no reason to suppose they would in general, especially if the GP isn’t working so well as a surrogate for the data-generating process (e.g. if it oversmooths and/or involves too little variability). Once each model is fitted by MLE then the models can be compared by LRT or AIC too - would Model 2 still be the favoured one?

    Response: We agree and have updated Table 1 so that it contains modal estimates from the sampling distributions (Page 16, Table 1). While we agree that there could be some value in reporting MLEs for these parameters and performing these other tests, this is not the main objective of our work. Moreover, for our confidence intervals and the modes of the sampling distributions, we have shown that our method is able to recover true parameter values in the synthetic data analysis (Pages S2–S8, Section S1), proving confidence in our methods and results. Finally, for the goodness of fit of the Gaussian process as a surrogate for the dynamical system (rather than the data-generating process, see Responses 4 and 5), this is straightforward to check by simply plotting and inspecting the Gaussian process, in which case the user of the method would work on refining the Gaussian process before fitting all their models, so there would be no major concern about the parameter values being captured once the user reaches this point of the method. It is also possible for the MLEs to try and fit the experimental data too

well while not trying to recover the underlying dynamical system, while our method can balance these demands because we combine two loss functions.
