# Supplementary material for: Computationally efficient mechanism discovery for cell invasion with uncertainty quantification
Source: PLoS Comput Biol. 2022 Nov 16;18(11):e1010599. doi: 10.1371/journal.pcbi.1010599 (PMC9710850; doi:10.1371/journal.pcbi.1010599)
Supplement: Attachment — Submitted filename: Response_to_Referees.pdf [file pcbi.1010599.s004.pdf]

Referee 1: Dr Ben Lambert

1. I thank the authors for their detailed resubmission and responses, which addressed all of my concerns. As an aside, I found the work that they have subsequently done on the impact of grid sizes on estimates really quite dramatic – that's quite a speed up – and it's interesting how robust the estimates are to these numerics.

   Response: We thank Dr Lambert for his time and enthusiastic response.

Referee 2

1. The authors have addressed all my questions and suggestions in the revised manuscript.

   Response: We thank Referee 2 for their time in reading our revised manuscript.

Referee 3

1. The authors have clearly gone to good lengths to address the comments from me and the other reviewers.

   Response: We thank Referee 3 for their positive report, and we now address their remaining questions.

2. I do have a remaining point relating to how the uncertainty quantification should be interpreted (which it seems key to be clear on given that this is a central focus of the manuscript). p10 of the revised manuscript directly addresses my points 4 and 5 along with their response "We do not attempt to mimic the precise data-generating process with our Gaussian process. Instead, we use the Gaussian process as a surrogate for the dynamical system that best describes the de-noised data-generating process". This is clearly explained but I'm still not clear on the rationale for that choice. Usually a parametric bootstrap seeks to mimic the data-generating process as closely as possible (including the noise structure of the observed data) so that, e.g., the CIs have a precise interpretation as uncertainty in the inferred parameters owing to sampling variability, i.e. having a finite number of noisy data points. If the parametric bootstrap is mimicking a de-noised process (albeit with some variability) then can the authors clarify how are CIs, and other inference, should instead be interpreted?

   Response: In the revised manuscript we now emphasise that Gaussian processes are an attractive model relative to other possible models (e.g. splines) since Gaussian processes are differentiable. This is very important when working with a PDE model so that we can estimate $u(x,t)$, $\partial u(x,t)/\partial t$, $\partial u(x,t)/\partial x$ and $\partial^2 u(x,t)/\partial x^2$, even in the presence of noise. We now mention this in the revised manuscript (Page 10, Paragraph 1).

   To address the question about the interpretation of uncertainty we added a brief discussion (also Page 10, Paragraph 1), noting that the de-noised results were estimated from the original noisy data. This means that noise is propagated from the data-generating process into our estimate of the underlying dynamical system. Therefore, the uncertainty that we describe is a consequence of the initial sampling variability in our experimental data. Therefore, all uncertainty and inference for this dynamical system is interpreted relative to the data-generating process (i.e. the experiments).
